# Rapid Modification of Proteins Using a Rapamycin-Inducible Tobacco Etch Virus Protease System

**DOI:** 10.1371/journal.pone.0007474

**Published:** 2009-10-15

**Authors:** Damian J. Williams, Henry L. Puhl, Stephen R. Ikeda

**Affiliations:** Laboratory of Molecular Physiology, National Institute on Alcohol Abuse and Alcoholism, National Institutes of Health, Bethesda, Maryland, United States of America; Mount Sinai School of Medicine, United States of America

## Abstract

**Background:**

The ability to disrupt the function of a specific protein on a rapid time scale provides a powerful tool for biomedical research. Specific proteases provide a potential method to selectively cleave a chosen protein, but rapid control of protease activity is difficult.

**Methodology/Principal Findings:**

A heterologous expression system for rapid target-directed proteolysis in mammalian cells was developed. The system consists of an inducible NIa protease from the tobacco etch virus (TEVp) and a chosen protein into which a TEVp substrate recognition sequence (TRS) has been inserted. Inducible activity was conferred to the TEVp using rapamycin-controlled TEVp fragment complementation. TEVp activity was assayed using a FRET-based reporter construct. TEVp expression was well tolerated by mammalian cells and complete cleavage of the substrate was possible. Cleavage at 37°C proceeded exponentially with a time constant of approximately 150 minutes. Attempts to improve cleavage efficiency were hampered by substantial background activity, which was attributed to inherent affinity between the TEVp fragments. A second TEVp assay, based on changes in inactivation of a modified K_V_3.4 channel, showed that functional properties of a channel can be using altered using an inducible TEVp system. Similar levels of background activity and variability were observed in both electrophysiological and FRET assays.

**Conclusions/Significance:**

The results suggested that an optimum level of TEVp expression leading to sufficient inducible activity (with minimal background activity) exists but the variability in expression levels between cells makes the present system rather impractical for single cell experiments. The system is likely to be more suitable for experiments in which the cell-to-cell variability is less of an issue; for example, in experiments involving large populations of cells.

## Introduction

The ability to ablate the function of a specific protein is a powerful tool for investigating protein function. Two approaches are commonly employed to achieve this goal. First, pharmacological agents, such as receptor antagonists, are used to acutely inhibit protein function. This approach is both rapid and, in some cases, selective. However, appropriately selective small molecule agents are often unavailable, especially for intracellular targets, and pharmacological effects are difficult to spatially constrain at the cellular level. Second, genetic approaches are used to reduce or eliminate protein expression by targeting the gene coding for the protein of interest (genetic knock-out) or interfering with gene transcription or translation (RNA interference, antisense RNA). These methods target protein synthesis and thus turnover of endogenous protein must occur before a phenotype is realized. During this delay, compensatory mechanisms can occur which confound data interpretation [1]–[4]. Genetic mechanisms, however, are broadly applicable if sequence information is available. Moreover, targeted expression strategies can be used to restrict modification to a small subset of cells in living animals. Thus, a method to modify the function of a specific protein on a rapid timescale (e.g.<1 hr) using both gene expression and pharmacological approaches would benefit from the advantages of both technologies.

A potential approach is to use a protease whose activity can be temporally controlled by small molecules and can specifically cleave the protein of interest at an identified site thereby altering or eliminating function. The tobacco etch virus NIa protease (TEVp) is a good candidate for this strategy as the canonical substrate recognition sequence (ENLYFQ/G) is sufficiently unique to ensure targeting specificity [5]. Moreover, TEVp has high catalytic activity [6], works over a broad pH range, and retains activity at temperatures from 4–37°C. These characteristics have led to the increasing *in vitro* use of TEVp in recombinant protein preparation, primarily for removing fusion tags from newly synthesized protein [7]. Consequently, high resolution structural information is available [8], [9] and efforts have been made to improve enzyme activity [6], [10] and solubility [11], [12].

Temporal control of TEVp enzyme activity, which is normally constitutive, can be conferred by using protein-fragment complementation (PFC), a technique originally developed to investigate protein-protein interactions [13]. In this system, a reporter protein is split into nonfunctional fragments and fused to potentially interacting proteins. Direct interaction between the protein partners brings the reporter protein fragments into close proximity resulting in assembly of a functional protein. Recently, TEVp has been used in a protein-fragment complementation assay to investigate protein-protein interactions [14]. In these studies, we used a rapamycin-mediated heterodimerization system combined with PFC to confer temporal control to TEVp activity. Split TEVp fragments were fused to either FK506 binding protein (FKBP12) or the FKBP12-rapamycin-binding domain of FRAP (FRB). Upon application of rapamycin, a cell permeable molecule, FKBP12 and FRB rapidly associate, resulting in reconstitution of TEVp enzyme activity. To quantify TEVp activity in living cells, we measured Förster resonance energy transfer (FRET) of reporter proteins comprised of fluorescent proteins linked together with a peptide containing a TEVp substrate recognition sequence (TRS). We also employed electrophysiological assays to explore the potential for acute post-translational modification of ion channels during patch clamp experiments. The results provide insights into both the potential and challenges of constructing genetically expressible systems for the rapid modification of targeted proteins in living cells.

## Results

### Quantification of TEVp enzyme activity in living cells using Förster resonance energy transfer measurements

**Figure 1:**
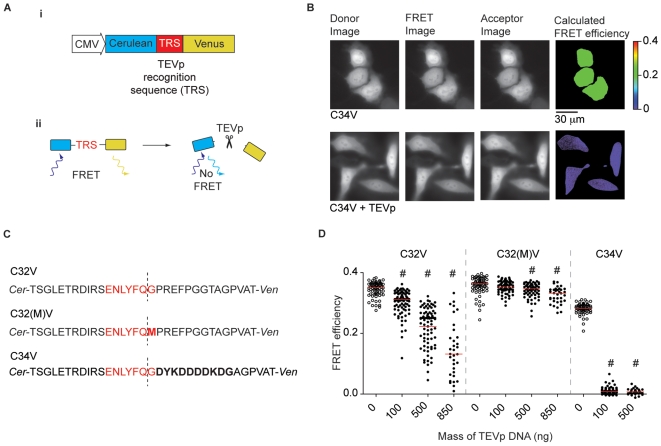
FRET-based assay of TEVp activity. *A*) (*i*) A schematic representation of the FRET sensor plasmid. (*ii*) Diagram showing the basis of the TEVp activity assay. *B*) Typical unprocessed donor, FRET and acceptor images and pseudocolored FRET efficiency images calculated from the intensities of the unprocessed images. Cells displaying high (C34V alone) and low (C34V + TEVp) FRET efficiencies are shown. *C*) Amino acid sequence of linkers between fluorescent proteins of the FRET sensor. The TEVp recognition sequence is shown in red and the cleavage site is marked with a dashed line. Differences between the linkers are shown in bold type. *D*) Scatter plot showing FRET efficiencies of individual cells transfected with 100 ng of one of the FRET sensor plasmid alone and with an increasing amount of TEVp plasmid vector. The median FRET efficiency is marked with a red bar. # represents a significant difference (*P*<0.05) between the median FRET efficiency of control cells transfected with sensor alone (open circles) and cells co-transfected with the amount of TEVp plasmid indicated (solid circles).

A FRET-based assay based on wide-field fluorescence microscopy was used to quantify TEVp activity. The genetically encoded substrate consisted of a donor, Cerulean [15], and an acceptor, Venus [16], fluorescent protein (FP) joined by a linker containing a TEVp recognition sequence (TRS; Fig. 1A). FRET was measured from sensitized acceptor emission using a modification of the 3-cube approach [17]–[19] and converted to FRET efficiency, as described previously [20]. This method also allows calculation of donor to acceptor ratio ([D]/[A]). For simple systems such as this, the measured or apparent FRET efficiency is a product of the intrinsic (or maximum) FRET efficiency and fractional occupancy of donor with acceptor. Thus, FRET efficiency represents a linear measurement of substrate cleavage that is instrument-independent (Fig. 1A *ii* and B).

As viral TEVp is expressed in plants, a codon-optimized (human) sequence was synthesized (GenBank accession # DQ516974) by a commercial source to optimize translation efficiency in mammalian cells. This sequence also contained a point mutation (S219V) introduced to prevent autolysis [6] and truncated C-terminus that removed a potentially inhibitory sequence [9]. Subsequently, three additional mutations (T17S, N68D, I77V) were introduced into the TEVp sequence to improve solubility [12]. To assess TEVp activity in living cells, various quantities of TEVp vector along with a reporter construct, C32V, were transfected into HeLa cells. C32V codes for a protein consisting of Cerulean and Venus linked via a 32-residue peptide that includes a canonical TEVp recognition sequence at the C-terminus (Fig. 1C). Following overnight incubation at 36°C, FRET efficiency for individual cells was determined (mean FRET efficiency determined on a pixel-by-pixel basis over the entire cell) and displayed as a scatter plot (Fig. 1D). We chose to graph the value for each cell as this format conveys the most information for data sets with moderate *n* values [21]. Moreover, for single cell assays (e.g., imaging and patch-clamp electrophysiology), the scatter in the data heavily influences experimental design. In the absence of TEVp expression, the FRET efficiency values were tightly clustered around a median of 35% (*n* = 71). For clarity, median FRET efficiencies measured in the presence of TEVp constructs were normalized to median FRET efficiencies measured from control cells transfected with the reporter construct alone. Raw FRET efficiencies are plotted in the Figures. Increasing quantities of TEVp cDNA produced progressively larger decreases in median FRET efficiency (Fig. 1D). In cells transfected with the highest mass of TEVp construct (850 ng), there was a large variation in FRET efficiencies and only a moderate reduction in median FRET efficiency (to 62% of the median FRET efficiency measured from control cells transfected with C32V alone; *n* = 33), which indicates incomplete substrate cleavage. In addition, the [D]/[A] increased from a median of 1.0 (interquartile range, IQR, 1.0–1.1; *n* = 34) to 2.0 (IQR 1.5–3.1; *P*<0.05; *n* = 33). This change suggested that the Venus species was degraded following cleavage. Preliminary experiments suggest the proteasomal degradation was involved as the [D]/[A] could be maintained near 1 if the cells were preincubated with MG-132, a cell permeant proteosome inhibitor (unpublished data).

In order to prevent possible inaccuracies in FRET calculations that result from large changes in [D]/[A], a methionine was inserted into the TRS at the P1′ position (C32[M]V; Fig. 1C). At this position, the resulting C-terminal cleavage product (i.e., Venus) should start with methionine, as with most naturally occurring proteins. Previous studies of TEVp substrate specificity, mostly performed *in vitro*, have indicated that the P1′ position is tolerant to all substitutions except proline [22]. Somewhat surprisingly, in cells transfected with 100 ng C32[M]V and 850 ng TEVp, there was only a small decrease in the median FRET efficiency (to 94% of the FRET efficiency measured from control cells transfected with C32[M]V alone; *n* = 35; Fig. 1D). This finding indicates that methionine at the P1′ position of the TRS strongly inhibited cleavage and that amino acid substitutions at this position may be more context sensitive than previously realized. Consequently, a third construct, termed C34V, which contained a FLAG® epitope (DYKDDDDKDG) located just downstream to the TRS was tested (C34V; Fig. 1C). There was a dramatic reduction in FRET efficiency in cells cotransfected with 100 ng TEVp (to 3% of the median FRET efficiency from cells transfected with C34V alone, *n* = 71, *n_control_* = 64; Fig. 1D). Moreover, there was little spread in these data, which indicates nearly complete substrate cleavage and the [D]/[A] ratio following cleavage remained near 1 (0.93, *n* = 71). Thus, C34V appeared to be a sensitive detector for TEVp activity and was used in all subsequent FRET-based experiments.

### Generation of regulable TEVp via protein fragment complementation

**Figure 2:**
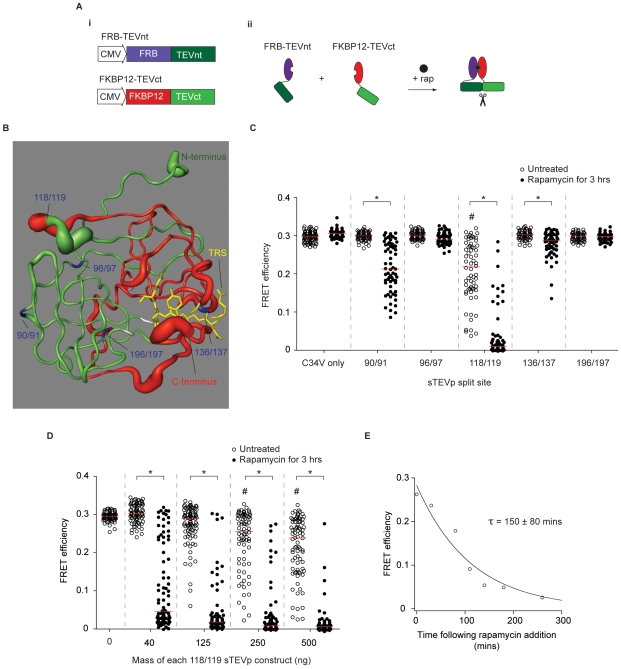
Development of an inducible TEVp using protein fragment complementation. *A*) (*i*) Plasmids encoding TEVp fragments (sTEVp-NT and sTEVp-CT) fused to rapamycin binding proteins (FRB and FKBP12). (*ii*) A representation of the rapamycin-induced complementation of sTEVp and reconstitution of protease activity. *B*) Crystal structure of TEVp (from [8]) represented as a cartoon showing ‘B-factor’. An increased diameter of the tube indicates a higher B-factor. The TRS of the substrate is shown in yellow and tested split sites are indicated in blue. The location of the residues required to catalyze proteolysis are shown in white. The two fragments that result from a 118/119 split are shown in green and red. *C*) Scatter plot showing FRET efficiencies of individual cells transfected with sTEVp constructs (split at the residues indicated). Cells were transfected with 150 ng of C34V alone, or cotransfected with 450 ng of each sTEVp plasmid. FRET efficiencies were measured from untreated cells and cells exposed to rapamycin for 3 hours. *D*) Scatter plot showing FRET efficiencies of individual cells transfected with different amounts of 118/119 sTEVp constructs. Measurements were taken from cells transfected with 100 ng of C34V alone, or cotransfected with the indicated amount of each sTEVp plasmid. FRET efficiencies were measured from untreated control cells and cells exposed to rapamycin for 3 hours. # represents a significant difference between control cells transfected with C34V alone and cells cotransfected with C34V and the sTEVp plasmids indicated. * represents a significant difference between cells transfected with the indicated sTEVp constructs in the absence of rapamycin (open circles) and cells exposed to 100 nM rapamycin for 3 hours (solid circles). *E*) A plot showing the median FRET efficiencies of different cells transfected with 300 ng of 118/119 sTEVp constructs at various time points after rapamycin addition. The time constant (τ) was calculated from a single-exponential curve fitted to the data (*n* = 15–30 cells at each time point).

To make a TEVp with inducible enzyme activity, two strategies were used: a PFC approach and by steric hindrance of the active site. For the PFC method, the TEVp open reading frame was split and the resulting N- and C-terminal fragments were fused to FKBP12 and FKBP12-binding protein (FRP), respectively (Fig. 2A). The choice of split sites was based on information derived from the crystal structure of TEVp (Fig. 2B) or the existing literature [14]. Split sites were chosen in domains with no clear secondary structure or regions in which a high degree of flexibility was inferred from the B-factor (atomic displacement parameter). To test each of the split sites, HeLa cells were cotransfected with 450 ng of both split TEVp (sTEVp) fragment plasmids and 100 ng C34V. Following overnight incubation to allow for protein expression, FRET efficiency measurements were made from cells exposed to 100 nM rapamycin for 3 hours at 37°C and untreated control cells. Rapamycin incubation had no effect on C34V FRET efficiency in the absence of TEVp fragment transfection (Fig. 2C). Of the five split sites tested, three produced a significant reduction in C34V FRET efficiency after rapamycin addition (90/91, 118/119, and 136/137; Fig. 2C). Cells transfected with 118/119 sTEVp (site from Wehr *et al.* [14]) produced the largest reduction in median FRET efficiency after rapamycin addition (to 3% of the median FRET efficiency measured from control cells transfected with C34V alone, *n* = 68, *n_control_* = 81), but there was also a significant reduction in FRET efficiency in the absence of rapamycin (to 83% of the FRET efficiency measured from control cells, *n* = 64). This reduction suggested that appreciable background TEVp activity (“leak”) was present when large quantities of 118/119 sTEVp were expressed.

The mass of 118/119 sTEVp construct used for transfection was adjusted to provide optimal sTEVp activity (i.e. minimum background TEVp activity and a high level of C34V cleavage after rapamycin addition). As shown in Fig. 2D, at low sTEVp expression levels (40 ng plasmid), there was little background activity but many cells displayed inefficient cleavage of C34V. As the amount of sTEVp used for transfection increased, an increase in both cleavage of C34V in control cells and cells exposed to rapamycin for 3 hours occurred. The optimum mass of sTEVp used for transfection was 125 ng. In the absence of rapamycin, in cells transfected with 125 ng sTEVp, there was a small decrease in median FRET efficiency (to 96% of the FRET efficiency measured from control cells transfected with C34V alone; *n* = 77, *n_control_* = 74). Following exposure to rapamycin for 3 hours, the median FRET efficiency decreased to 3% of that of control cells (*n* = 68). A similar level of background activity occurred in cells transfected with 118/119 sTEVp constructs lacking FRB and FKBP12 domains (Fig. S1A). These results suggest that the background activity was caused by spontaneous association of the TEVp fragments rather than association of the FRB and FKBP12 domains in the absence of rapamycin.

### Cellular kinetics of TEVp action

To investigate the time course of sTEVp activity, cells were transfected with 300 ng of each of the 118/119 sTEVp constructs and 100 ng C34V. FRET efficiencies from different cells were measured before rapamycin addition and at regular intervals for 4 hours in the presence of rapamycin at 37°C. A single exponential curve was fitted to the median FRET efficiency at each time point (*n* = 15–30) using nonlinear regression. The time constant (τ) of the fitted line was 150 minutes (Fig. 2E). TEVp produced in bacteria have a folding defect that reduces activity when grown at 37°C (D.S. Waugh, personal communication) and thus a similar experiment was run at 33°C. However, the temperature reduction had little effect on the rate of proteolysis (the τ was 170 minutes, *n* = 23–39; Fig. S1B) indicating that differences between the constructs (e.g., introduced solubility mutations) or cell type (mammalian cells vs. *E. coli*) affected the relationship between the occurrence of the folding defect and temperature.

A simple model was employed to provide a framework for further optimization:
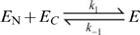
(1)

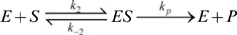
(2)where *E_N_* and *E_C_* are the N- and C-terminus fragments of TEVp, *E* is the reconstituted or functional enzyme (TEVp), *S* is the substrate (C34V), *ES* the enzyme-substrate complex, and *P* the products of hydrolysis. The rate constants (*k*) are as indicated. It was assumed that the formation of the functional enzyme occurs rapidly once the E_N_-E_C_ complex was formed (Equation 1) and that the usual assumptions [23] underlying the Michaelis-Menten formalism were made. From the time course data shown in Fig. 2E, we can infer that mechanisms underlying TEVp reconstitution (e.g., diffusion of rapamycin into the cell, formation of E_N_-E_C_ complex, and folding to form the functional enzyme) were relatively rapid on this time scale (hundreds of minutes) as the substrate concentration decreased monotonically with little evidence of delay. As the time course was well approximated by a single exponential function, we also infer that [S]≪*K_M_* of the reconstituted TEVp.

In parallel with the PFC strategy, we undertook a second approach to construct an inducible TEVp system. For this method, the idea was to occlude access to the TEVp active site using a point mutant of the FKBP12 protein termed Fm [24]. When expressed, Fm forms homodimers that can subsequently be disrupted by the addition of rapamycin. By fusing Fm proteins fused to the N- and C-termini of TEVp, it was hypothesized that TEVp activity would be ablated by steric hindrance of the catalytic site. Addition of rapamycin would then restore enzyme activity by disrupting the Fm-Fm interaction and reestablishing access to the active site. In principle, this approach offered potential advantages over a PFC strategy. First, delays arising from complementation would not occur, a problem that would manifest at lower concentrations of enzyme (see Equation 1). Second, complementation often decreases the intrinsic activity of enzymes and thus a “native” enzyme conformation was preferred. Unfortunately, although a large number of Fm-TEVp constructs (see “Supplemental methods S1”) were generated, all retained sufficient constitutive activity (Fig. S2) to render them inappropriate for the intended purpose. Consequently, we focused on refining the PFC strategy to create an effective inducible TEVp system.

### Unitary split TEVp constructs

**Figure 3:**
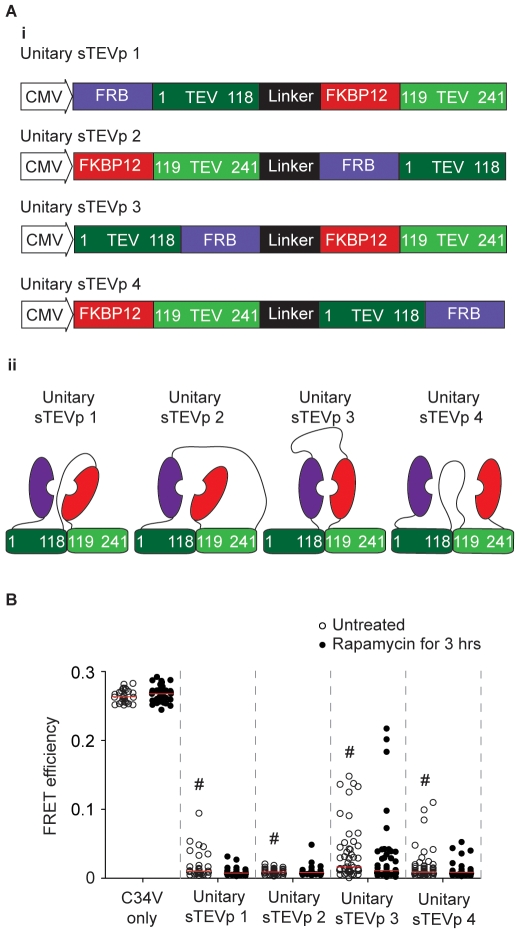
Unitary sTEVp constructs have a high background protease activity. *A*) (*i*) Unitary sTEVp plasmids. Locations of the linker and the FKBP12 and FRB proteins in relation to the TEVp fragments are shown. The amino acids at the N- and C-termini of the TEVp fragments are labeled corresponding to their location in the full length TEVp. *ii*) Schematic diagram of the unitary sTEVp proteins. *B*) Scatter plot showing FRET efficiencies of individual cells transfected with unitary sTEVp constructs. Cells were transfected with 100 ng of C34V and 600 ng of the unitary sTEVp plasmid. For each construct, FRET efficiencies were measured from untreated cells (open circles) and cells treated with rapamycin for 3 hours (solid circles). # represents a significant difference between the median FRET efficiency of control cells transfected with C34V alone and cells cotransfected with C34V and the unitary sTEVp plasmid indicated.

Because our initial sTEVp constructs displayed slow proteolysis rates following rapamycin addition, we employed several strategies in an attempt to improve the speed. The first approach utilized unitary constructs in which the sTEVp fragments were joined together by a flexible peptide linker. We reasoned that if the association of separate sTEVp fragments was rate limiting (Equation 1), the proximity afforded by these constructs (i.e., an increase in effective concentration) would accelerate the process. Unitary constructs were created with the linker or FRB/FKBP12 proteins located in different positions (Fig. 3A). The constructs fell into two broad classes: i) large insertions into the split site (constructs 1 and 3) or, ii) circularly permuted (swapping of the N- and C-termini) with or without insertions (constructs 2 and 4). It was hoped that such dramatic disruptions to the primary sequence would prevent enzymatic activity until addition of rapamycin facilitated reconstitution of the active species. Similar strategies have been used successfully for constructing rapamycin-inducible luciferase activity [25]. However, in cells transfected with 300 ng of any of the unitary constructs, there was a large decrease in median FRET efficiency in both the absence and presence of rapamycin (to <7% of the median FRET efficiency measured from control cells transfected with C34V alone, *n* = 28–54). These results indicated that the unitary sTEVp constructs possessed substantial enzymatic activity in the absence of rapamycin. Given the severe disruption of the primary sequence by large insertions with predicted secondary structure, the retention of enzymatic activity was surprising and suggested a propensity of the TEVp fragments to spontaneously re-associate. The inability to modulate TEVp activity with rapamycin rendered this approach unsuitable.

### cis-based sTEVp constructs

**Figure 4:**
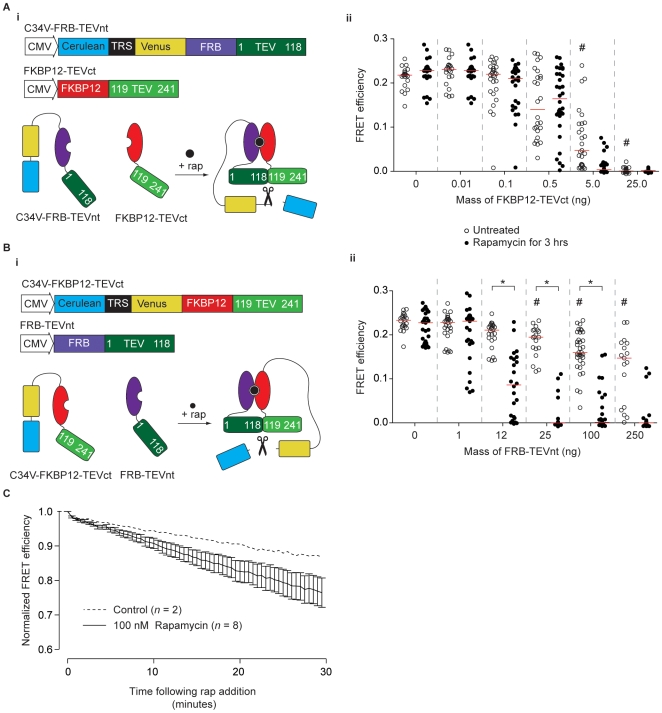
Fusion of the substrate to one sTEVp fragment increased background protease activity with no substantial increase in inducible protease activity. *A*) *cis*-sTEVp A. (*i*) Plasmid expressing C34V fused to FRB-sTEVp. FKBP12-sTEVp was expressed from a second plasmid (*ii*) Scatter plot showing FRET efficiencies of cells transfected with 300 ng of C34V-FRB-sTEVp plasmid alone and with increasing amounts of FKBP12-sTEVp. FRET efficiencies were measured in control cells and in cells exposed to rapamycin for 3 hours. # represents a significant difference of median FRET efficiencies between the control cells transfected with C34V-FRB-sTEVp alone (first column) and cells cotransfected with the mass of FKBP12-sTEVp indicated, in the absence of rapamycin. * represents a significant difference between median FRET efficiency measured from cells in the absence of rapamycin and cells treated with 100 nM rapamycin for 3 hours. *B*) *cis*-sTEVp B. (*i*) Plasmid map and schematic diagram showing C34V fused to FKBP12-sTEVp. FRB-sTEVp-NT was expressed from a second plasmid. (*ii*) Scatter plot of FRET efficiencies of cells transfected with 300 ng of C34V-FKBP12-sTEVp-CT plasmid alone and with an increasing amount of FRB-sTEVp-NT. Details of the graph are equivalent to those described in part (*A*), above. *C*) Time course of *cis*-sTEVp Construct B activity. Cells were transfected with 300 ng of C34V-FKBP12-sTEVp-CT and 25 ng of FRB-sTEVp-NT. FRET efficiencies were measured from the same cells, every 30 s and were normalized to the first FRET efficiency measurement, before 100 nM rapamycin was added. The blue line represents measurements taken from control cells to which no rapamycin was added. Mean ± SE FRET efficiency data plotted.

TEVp is expressed from the TEV genome as part of a large polyprotein, which TEVp cleaves in a *cis* configuration to release separate viral proteins [26]. This tethered arrangement provides a very high effective substrate concentration that increases enzyme velocity (equation 2). Two *cis*-sTEVp constructs were produced by fusing one fragment of sTEVp to C34V and expressing the second fragment of sTEVp from a separate plasmid (Fig. 4A *i*, B*i*). The advantage of this configuration is that reconstituted enzyme is exposed to a consistent and high effective concentration of substrate regardless of expression levels. The disadvantage is that the targeted molecule requires a larger and more complex modification. Optimum inducible protease activity for each of the *cis*-sTEVp construct was determined by titrating the amount of free sTEVp construct against a fixed amount (300 ng) of the C34V-fused construct. In cells transfected with C34V-FRB-TEVnt (Fig. 4A *i*), there was a decrease in FRET efficiency as the amount of FKBP12-TEVct was increased over the range 0.01–25 ng (Fig. 4A *ii*). At the highest concentration tested (25 ng), cleavage was nearly complete as indicated by a negligible FRET efficiency. In all transfections of C34V-FRB-TEVnt, rapamycin did not cause a significant change in FRET efficiency indicating minimal inducible TEVp activity. A second set of *cis*-sTEVp constructs comprised of C34V-FKBP12-TEVct and FRB-TEVnt (Fig. 4B *i*) were also tested. In the absence of rapamycin, cells transfected with ≥25 ng FRB-TEVnt had a significant decrease in FRET efficiency compared to cells transfected with 300 ng C34V-FKBP12-TEVct alone (Fig. 4B *ii*). These results were indicative of constitutive protease activity at higher FRB-TEVnt concentrations. Addition of rapamycin to cells transfected with 12, 25, and 100 ng FRB-TEVnt caused a significant reduction in FRET efficiency (Fig. 4B *ii*). Optimum inducible TEVp activity occurred in cells transfected with 25 ng FRB-TEVnt. In these cells, there was little background protease activity: the median FRET efficiency in the absence of rapamycin was 84% of the median FRET efficiency measured from control cells transfected with C34V-FKBP12-TEVct alone (*n* = 27, *n_control_* = 19). Following exposure to rapamycin for 3 hours, the median FRET efficiency fell to 0% of that of the control cells (*n* = 20), which suggested there was a high level of inducible protease activity. The concentration range over which there was optimal *cis*-sTEVp activity was narrow: at higher concentrations of FRB-TEVnt constitutive activity was apparent while at lower concentrations there was insufficient inducible activity.

To investigate the rate of protease activity under the desired experimental conditions (i.e., those mimicking patch-clamp experiments), cells were transfected with 25 ng FRB-TEVnt construct plus 300 ng C34V-FKBP12-TEVct. FRET efficiencies were measured from the same cells during a control period and for 30 minutes after the addition of 100 nM rapamycin. These experiments were performed on the microscope stage at room temperature. FRET efficiencies were normalized to measurements taken in the control period. After exposure to rapamycin for 20 minutes, the mean FRET efficiency decreased by approximately 20% (Fig. 4C). In control cells (not exposed to rapamycin), there was a small reduction in FRET efficiency (a reduction of approximately 10%; perhaps indicative of a photobleaching of acceptor (Venus) or a moderate level of background TEVp activity.

### Targeting of sTEVp and substrate to the plasma membrane

**Figure 5:**
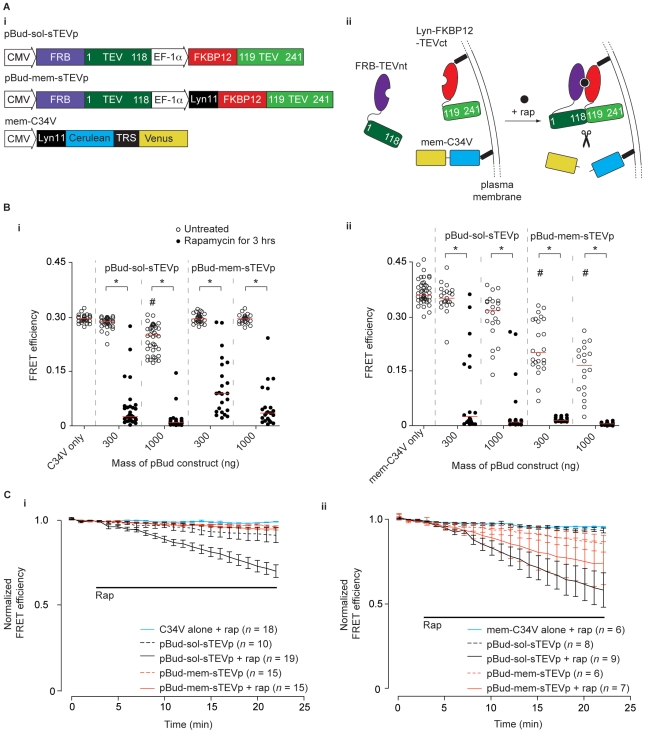
Targeting of one sTEVp fragment and C34V to the membrane increased both inducible and background activity of sTEVp. *A*) (*i*) A single plasmid was used to express both sTEVp fragments. pBud-mem-sTEVp contains a Lyn11 membrane-targeting sequence fused to the N-terminus of FKBP12-sTEVp-CT. Mem-C34V has a Lyn11 sequence fused to the N-terminus of C34V. (*ii*) Schematic diagram showing localization of the Lyn11-modified sTEVp fragment and mem-C34V. *B*) Scatter plots showing FRET efficiencies of cells transfected with (*i*) soluble C34V and (*ii*) membrane-targeted C34V alone, and with 0.3 µg and 1 µg of pBud-sol-sTEVp or pBud-mem-sTEVp DNA. # represents a significant difference in median FRET efficiencies between control cells transfected with C34V alone and cells cotransfected with the indicated mass of pBud construct. * represents a significant difference of median FRET efficiency between untreated cells and cells exposed to 100 nM rapamycin for 3 hours, for a given mass of pBud-sTEVp plasmid. *C*) Time course of sTEVp activity. Cells transfected with 100 ng of (*i*) soluble C34V or (*ii*) membrane-targeted C34V alone, and cotransfected with 1000 ng of pBud-sol-sTEVp or pBud-mem-sTEVp. FRET efficiencies were measured every 60 seconds. Where indicated, rapamycin was added 3 minutes after the start of the experiment. FRET efficiencies were normalized to the mean FRET efficiencies measured before the addition of rapamycin. Mean ± SE FRET efficiency data plotted.

The high effective substrate concentration in the previous two *cis*-configuration systems revealed an undesirable propensity of sTEVp to reconstitute in the absence of rapamycin. Although this property was evident in the *trans*-proteolysis experiments (Fig. 2D), the magnitude of rapamycin-mediated proteolysis was adequate over a wide range of concentrations. Thus, an intermediate solution was sought to increase effective substrate concentration, and hence enzyme velocity, while avoiding overwhelming constitutive activity. To this end, one fragment of TEVp and the substrate were targeted to the plasma membrane. Both fragments of sTEVp were cloned into a single vector (pBud-sol-sTEVp or pBud-mem-sTEVp; Fig. 5A *i*), with each open reading frame driven by a different promoter, to ensure that the sTEVp fragments were expressed at a consistent ratio. For localization to the plasma membrane, a targeting motif (N-terminal myristoylation and palmitoylation) from the protein *Lyn* [27] was fused to the N-terminus of C34V (mem-C34V) and the N-terminus of FKBP12-TEVct (pBud-mem-sTEVp; Fig. 5A *i, ii*).

Experiments were carried out to assess cleavage of (cytosolic) C34V by either cytosolic or membrane-targeted sTEVp. As shown in Fig. 5B *i*, the median FRET efficiency of cells transfected with 300 ng of pBud-sol-sTEVp and 100 ng of C34V was 97% of the FRET efficiency measured from control cells expressing C34V alone (*P*>0.05, *n* = 30, *n_control_* = 36). In the presence of rapamycin, the median FRET efficiency of cells transfected with 300 ng pBud-sol-STEVp decreased to 9% of that of control cells (*n* = 33) and varied substantially from cell to cell. Cells were transfected with a higher quantity of pBud-sol-sTEVp (1000 ng) in an attempt to improve the amount of C34V cleavage. In these cells, there was small but significant decrease in FRET efficiency in the absence of rapamycin (84% of the control median FRET efficiency, *n* = 40) compared with cells expressing C34V alone (see above). In the presence of rapamycin, the FRET efficiency of cells transfected with 1000 ng of pBud-sol-sTEVp decreased to 3% of the control median FRET efficiency (*n* = 33) with little cell-to-cell variability. This result indicated that at higher concentrations of pBud-sol-sTEVp, there was an increase in inducible protease activity, but this was accompanied by increased background activity.

The effect of targeting sTEVp to the membrane on cleavage of cytosolic C34V was investigated (Fig. 5B *i*, right). There was no significant difference in median FRET efficiency between control cells transfected with 100 ng C34V alone and cells co-transfected with either quantity of pBud-mem-sTEVp (the median FRET efficiency was 100% of that of the control, for both 300 ng, and 1000 ng pBud-mem-sTEVp; *n* = 27 and 23 respectively). Following treatment with rapamycin, the median FRET efficiency decreased to 30% of that of the control cells (*n* = 23) for cells transfected with 300 ng of pBud-mem-sTEV, and 12% of that of the control cells (*n* = 24) for cells transfected with 1000 ng of pBud-mem-sTEV. These results show that for the soluble C34V, the membrane-bound sTEVp has a lower amount of background activity, but lower inducible protease efficiency than the cytosolic sTEVp.

Similar pBud-sTEVp experiments were repeated using the plasma membrane-targeted substrate, mem-C34V (Fig. 5B *ii*). In cells transfected with 300 ng pBud-sol-sTEVp and 100 ng of mem-C34V, the median FRET efficiency in the absence of rapamycin was 98% of the FRET efficiency measured from control cells transfected with mem-C34V alone (*n* = 21, *n_control_* = 45). In cells transfected with 1000 ng pBud-sol-sTEVp, the median FRET efficiency was 89% of that of control cells (*n* = 22). In the presence of rapamycin, there was very large decrease in FRET efficiency in both cells transfected with 300 ng pBud-sol-sTEVp (to 7% of control median FRET efficiency; *n* = 23) and 1000 ng of pBud-sol-sTEVp (to 3% of control median FRET efficiency; *n* = 18), although a number of cells displayed variable FRET efficiencies. These results suggest that the amount of inducible cleavage by sol-sTEVp of mem-C34V was high (with the exception of a few cells).

In cells transfected with pBud-mem-sTEVp, efficient cleavage of mem-C34V was accompanied by significant background activity. In the absence of rapamycin, the median FRET efficiency of cells transfected with 300 ng pBud-mem-sTEVp was 56% of that of control cells (*n* = 24); in cells transfected with 1000 ng of pBud-mem-sTEVp the median FRET efficiency was 46% of that of control cells (*n* = 18). In the presence of rapamycin for 3 hours, the median FRET efficiency was ≤4% of that of control cells (*n* = 20 and 23, for 300 ng and 1000 ng of pBud-mem-sTEVp, respectively).

To investigate the rate of the sTEVp and mem-sTEVp activity, cells were transfected with 1000 ng of one of the pBud-sTEVp constructs and 100 ng of C34V or mem-C34V. FRET efficiencies were measured from the same cells during a 2-minute control period and for 20 minutes after the addition of 100 nM rapamycin on the microscope stage at room temperature. FRET efficiencies were normalized to measurements taken in the control period. In cells transfected with pBud-sol-sTEVp, the mean FRET efficiency decreased by approximately 30% over 20 minutes after rapamycin addition (Fig. 5C *i*). The change in FRET efficiency varied widely between cells (see Fig. S3 for the traces of time courses from individual cells). In the cells transfected with pBud-sol-sTEVp but with no rapamycin added, there was a small reduction in FRET efficiency (8% reduction from normalized control FRET measurements, *n* = 10) over 20 minutes. This reduction was mainly due to a single cell with a large change in FRET efficiency (Fig. S3A *ii*). In cells transfected with C34V alone, or cotransfected with pBud-mem-sTEVp, there was no obvious change in FRET efficiency over the duration of the experiment. Changes in FRET efficiencies after rapamycin addition varied widely in cells transfected with mem-C34V (Fig. 5C *ii*; Fig. S3). Cells transfected with pBud-mem-sTEVp or pBud-sol-sTEVp showed substantial but variable decreases in FRET efficiencies over 20 minutes (a 25% and 40% reduction from normalized control FRET efficiency measurements, for pBud-mem-sTEVp and pBud-sol-sTEVp, respectively). The results were complicated by high levels of background activity (indicated by low FRET efficiencies in control measurements) in some cells transfected with pBud-mem-sTEVp. Reliable measurement of very low FRET efficiencies is difficult, and these inaccuracies are amplified by normalization. For this reason, cells with control FRET efficiencies of less than 0.05 (2/8 cells for control recordings, 5/12 cells for recordings in the presence of rapamycin) were not included in the results displayed in Fig. 5C *ii*.

### An electrophysiological assay for sTEVp activity

**Figure 6:**
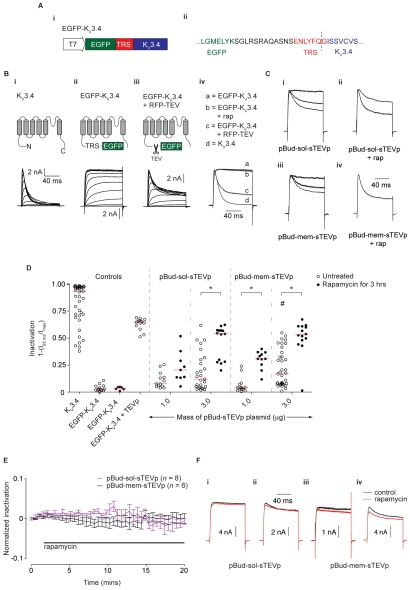
An electrophysiological assay of TEVp activity based on changes in inactivation of a modified K_V_3.4 channel. *A)* TEVp activity sensor (EGFP-K_V_3.4) consisted of an EGFP moiety fused to the N-terminus of a human K_V_ 3.4 subunit via a TRS. (*i*) EGFP-K_V_3.4 RNA was transcribed from a plasmid containing a T7 promoter. (*ii*) Amino acid sequence of the TRS region of EGFP-K_V_3.4. The C-terminus of EGFP is shown in green, the TRS in red, and the N-terminus of K_V_3.4 shown in blue. The cleavage site is marked with a dashed line. *B*) Schematic diagrams of K_V_3.4 constructs and current traces showing the basis of the K_V_3.4-based assay. Currents were recorded from HEK-293 cells held at −80 mV and stepped to command voltages (−80 mV to +60 mV, in 10 mV increments) for 100 ms. (*i*) Cells transfected with 500 ng wild-type K_V_3.4 RNA displayed strongly inactivating currents. (*ii*) Inactivation was blocked in cells transfected with 500 ng EGFP-K_V_3.4. (*iii*) Cleavage of EGFP from K_V_3.4 resulted in a partial recovery of inactivation in cells cotransfected with 1000 ng EGFP-K_V_3.4 RNA and 2 µg RFP-TEVp DNA. (*iv)* Current traces from different cells held at −80 mV and stepped to +50 mV for 100 ms. Currents were normalized to the same peak amplitude. Representative traces from cells transfected with (a) EGFP-K_V_3.4 RNA (b) EGFP-K_V_3.4 RNA + rap for 3 hours (c) EGFP-K_V_3.4 RNA + RFP-TEVp DNA (d) wild-type K_V_3.4 RNA. *C*) Typical current traces showing different levels of inactivation (normalized to the same peak amplitude) from different cells transfected with 1000 ng EGFP-K_V_3.4+ (*i*) 3 µg pBud-sol-sTEVp DNA (*ii*) 3 µg pBud-sol-sTEVp DNA+100 nM rap for 3 hours (*iii*) 3 µg pBud-mem-sTEVp (*iv*) pBud-mem-sTEVp+100 nM rap for 3 hours. *D*) Scatter plot showing the amount of current inactivation from cells transfected with the constructs described above. Inactivation was calculated using the amplitude of the current 95 ms after the start of the voltage step (I_95__ms_) and the peak current in the first 50 ms of the voltage step (I_max_); see *Methods* for details. # represents a significant difference in median FRET efficiency, between the cells transfected with EGFP-K_V_3.4 alone (second column) and the cells transfected with the indicated pBud-sTEVp, in the absence of rapamycin. * represents a significant difference between the median FRET efficiency from cells in the absence of rapamycin and cells treated with 100 nM rapamycin for 3 hours, for a given amount of pBud-sTEVp. *E*) Plot showing the degree of current inactivation from cells transfected with 3 µg pBud-sTEVp after addition of 100 nM rapamycin. Recordings were made every 10 s (30 s intervals plotted for clarity) and inactivation was normalized to control currents recorded in the first 2 minutes, before rapamycin addition. Mean ± SE data plotted. *F*) Representative traces from cells transfected with pBud-sTEVp before (black trace) and after addition of rapamycin for 15 minutes (red trace). Examples of cells displaying currents with no inactivation (*i, iii*) and clear inactivation (*ii*, *iv*) in the control recordings are shown.

An inducible TEVp system suitable for use in electrophysiological experiments would be desirable. For this reason, an assay in which inducible TEVp activity could be evaluated in the conditions and on the time-scale of an acute electrophysiological experiment was developed. For the assay, a TEVp substrate consisting of EGFP fused to the N-terminus of a K_V_3.4 subunit via a TRS (EGFP-K_V_3.4; Fig. 6A) was transfected into HEK-293 cells and currents through the modified K^+^ channel were monitored using whole-cell patch-clamp recording techniques. TEVp activity was determined by measuring changes in inactivation of the whole-cell current that occurred following TEVp-mediated cleavage of EGFP from the K_V_3.4 subunit. For these experiments, heterologous expression of K_V_3.4 constructs was achieved by electroporation of HEK-293 with RNA transcribed *in vitro*. This method provided high transfection efficiency and a more uniform level of K_V_3.4 expression than the PEI method used in the FRET experiments.

In cells transfected with 500 ng of wild-type K_V_3.4 RNA, voltage-dependent, strongly inactivating currents were elicited when the cells were held at −80 mV and stepped to command voltages (−80 mV to +60 mV, in 10 mV increments) for 100 ms (Fig. 6B *i*,). In cells transfected with 500 ng EGFP-K_V_3.4 RNA, sustained, non-inactivating, voltage-dependent currents were elicited by the same voltage protocols as above (Fig. 6B *ii*). When 2 µg of RFP-TEVp DNA was co-transfected with 1 µg EGFP-K_V_3.4 RNA (Fig. 6B *iii*), there was substantial (but incomplete) recovery of rapid, voltage-dependent inactivation. These results are consistent that idea that the rapid ‘N-type’ current inactivation of the wild-type K_V_3.4 channel (due to entry of the N-terminal domain into the open channel pore; 28) was prevented by the bulky EGFP domain fused to the N-terminus of K_V_3.4 in EGFP-K_V_3.4. Cleavage of EGFP from the modified K_V_3.4 subunit partially restored N-type inactivation. TEVp activity could therefore be assessed by measuring changes in current inactivation in cells expressing EGFP-K_V_3.4 and the TEVp construct of interest.

Inactivation (and hence TEVp activity) was quantified from currents elicited in cells held at −80 mV and stepped to +50 mV for 100 ms (Fig. 6B *iv*). This voltage protocol generated currents with robust inactivation that was insensitive to small changes in voltage. A non-inactivating current of <0.3 nA, likely to be caused by endogenous voltage-gated K^+^ or Cl^−^ channels [29], was elicited by the −80 to +50 mV step in untransfected HEK-293 cells (data not shown). For this reason, only currents with peak amplitudes >1.5 nA were used for inactivation calculations. The ‘degree of inactivation’ was calculated using the equation 1-(*I*_95ms_/*I*_max_), where *I*_95ms_ is the amplitude of the current 95 ms after the start of the voltage step, and *I*_max_ is the peak current during the first 50 ms of the voltage step. Quantification of current inactivation from control experiments (described above; Fig 6D, left side) shows the working range of inactivation in this assay: from a median of 0.03 (*n* = 13) in cells transfected with EGFP-K_V_3.4 alone (no inactivation/TEVp activity) to a median of 0.65 (*n* = 14) in cells transfected with EGFP-K_V_3.4 and RFP-TEVp (maximum inactivation/TEVp activity).

Inducible TEVp activity was assessed in cells transfected with either 1 µg or 3 µg of pBud-sol-sTEVp DNA, and 1 µg EGFP-K_V_3.4. In cells transfected with 1 µg of pBud-sol-sTEVp, the median inactivation (0.08; *n* = 13) was not significantly different from cells transfected with EGFP-K_V_3.4 alone (0.03; *n* = 13; Fig. 6D). Addition of 100 nM rapamycin for 3 hours did not cause a significant increase in median inactivation (0.20; *n* = 9). These results indicate that transfection of 1 µg pBud-sol-sTEVp did not provide high levels of inducible TEVp activity. In control cells transfected with an increased mass of pBud-sol-sTEVp DNA (3 µg), the median inactivation was 0.12 (*n* = 27) with many cells showing clear inactivation in the absence of rapamycin (Fig. 6C *i* and D), suggesting background protease activity was present. Following addition of rapamycin for 3 hours, inactivation increased (median inactivation = 0.54; *n* = 14), but the level of inactivation varied substantially between cells (Fig. 6C *ii* and D).

Because of the variable level of inducible protease activity provided by pBud-sol-sTEVp, experiments were carried out using pBud-mem-TEVp. The increase in apparent TEVp concentration conferred by membrane targeting was predicted to increase the level of substrate cleavage. In cells transfected with 1 µg pBud-mem-sTEVp, the median inactivation was 0.04 (*n* = 14), which was not significantly different from cells transfected with 1000 ng EGFP-K_V_3.4 RNA alone (Fig. 6D). In the presence of rapamycin, the median inactivation of currents from cells transfected with 1 µg pBud-mem-sTEVp significantly increased to 0.31 (*n* = 11). These results indicate that in cells transfected with a low quantity of pBud-mem-sTEVp, there is little background activity, but there was only a modest amount of inducible sTEVp activity. In order to improve the amount of cleavage, cells were transfected with 3 µg of pBud-mem-sTEVp. In these cells, inactivation varied highly from cell to cell, and the median inactivation significantly increased compared to cells expressing EGFP-K_V_3.4 alone (median inactivation = 0.17; *n* = 36; Fig. 6C *iii*, D). In the presence of rapamycin, there was a large increase in median inactivation with low variability between cells (median inactivation = 0.53; *n* = 14; Fig. 6C *iv*, D). These results indicate that expression of higher quantities of membrane-bound sTEVp increase inducible TEVp activity but are also associated with an increase in background activity.

To ascertain whether adequate sTEVp cleavage occurs on a timescale suitable for acute electrophysiological experiments, the time course of sTEVp activity was determined in HEK-293 cells co-transfected with 1 µg of EGFP-K_V_3.4 RNA and 3 µg of either pBud-sol-sTEVp or pBud-mem-sTEVp. Recordings were made from cells in a 2-minute control period and for 18 minutes following the addition of 100 nM rapamycin. Inactivation was normalized to the mean inactivation in the control period. In cells transfected with either pBud-sol-sTEVp or pBud-mem-sTEVp, the average inactivation changed by <1% (*n* = 6 and 8) over the duration of rapamycin exposure (Fig. 6E). Recordings were made from cells with and without basal inactivation visible in the control period (Fig. 6F), but addition of rapamycin did not augment inactivation in either case.

## Discussion

In this investigation, we have shown that protein function can be altered using a rapamycin-inducible TEVp and a TRS inserted into a protein of interest. The system worked well on a time scale of 3 hours, but the low activity of the sTEVp resulted in insufficient cleavage of the target protein on a shorter time scale (< 20 min). This study has identified a number of necessary considerations and problems encountered when using an inducible TEVp system.

### Context and sequence of the TRS

In addition to choosing a location of the TRS so that its cleavage will have the desired effect on the function of the protein of interest, this study suggests that cleavage efficiency is very dependent on the context and sequence of the TRS. Cleavage efficiency of the canonical TRS was greatly increased when a FLAG epitope sequence was inserted downstream of the TRS (Fig. 2). The reason for this improvement is unknown, but the charged nature of the FLAG sequence may improve access to the TRS. A previous study has suggested that many different residues, including methionine, can occupy the P1′ position of the TRS without a significant effect on cleavage efficiency [22]. However, in our study, insertion of methionine into the P1′ position (C32[M]V) strongly inhibited cleavage. These results suggest that it is difficult to predict efficient cleavage of the TRS and optimization of the TRS for each target protein is advantageous.

Evaluation of the efficiency of TRS cleavage in EGFP-K_V_3.4 is complicated by unknown effects of N-terminal modification on channel inactivation. Cells transfected with both EGFP-K_V_3.4 and full-length TEVp displayed approximately 70% of the current inactivation from cells transfected with wild-type K_V_3.4 alone. Given that inactivation of wild-type K_V_3.4 requires only one (of a possible four) functional N-terminus [30], it is possible that this incomplete inactivation represent low TRS cleavage. Alternatively, cleavage may be efficient but the presence of any remaining fused EGFP domains prevents complete inactivation.

### Low catalytic efficiency of sTEVp

The limited cleavage of C34V or EGFP-K_V_3.4 over 20 minutes suggests that sTEVp has a low catalytic efficiency. Given that Wehr *et al.* [14] found that the 118/119 sTEVp had 43% of the activity of the wild-type TEVp, it is likely that fragment complementation results in reduced enzyme efficiency. A number of other complemented enzymes have reduced activity [31]–[33] (although exceptions exist; e.g., [34], [35]. It is important to consider that wild-type TEVp may have an intrinsically low catalytic efficiency. The *cis* arrangement of TEVp and substrate in the viral polyprotein provides high apparent concentrations of enzyme. In this configuration, a very efficient enzyme may not be necessary for the requisite level of substrate cleavage. Sufficient enzyme activity is dependent on the nature of the experiment and is difficult to predict from the published values of K_cat_ and K_M_ for TEVp [6], [11]. These parameters are dependent on the substrate and experimental conditions, which were very different to those in our study.

### Background activity of sTEVp

In our experiments, attempts to overcome the low catalytic efficiency of the sTEVp were based on increasing the actual or apparent concentration of complemented sTEVp. While this method increased substrate cleavage, its usefulness was hindered by an increase in TEVp activity in the absence of rapamycin. Successful protein complementation requires that nonfunctional fragments of TEVp have a low affinity, and therefore the probability of fragments associating and reconstituting a functional enzyme at low concentrations is minimal. Results here are consistent with significant affinity between TEVp fragments, which results in a fraction of TEVp fragments that exist as functional, complemented enzymes. Cleavage of substrate by this fraction of enzyme is evident when the total concentration of sTEVp fragments (Fig. 2) or the apparent enzyme concentration is increased (*cis* constructs and membrane-targeted constructs; Fig. 4 and 5). In these examples, for a given amount of TEVp fragments, the proportion of the TEVp fragments that exist as functional proteases is not predicted to change. In the unitary constructs (Fig. 3), where the fraction of spontaneously complemented TEVp fragments is predicted to be higher, clear background activity is also visible. This type of ‘spontaneous’ refolding/re-association and reconstitution of catalysis of split enzyme fragments at relatively low concentrations is well established [36] and background activity of other split enzyme complement systems have been attributed to the high affinity between fragments [25], [37]. Assuming that our 118/119 sTEVp has significant affinity, any attempt to overcome the low catalytic efficiency by increasing apparent/actual sTEVp concentration is likely to result in a concomitant increase in background activity. An alternative method to improve substrate cleavage by increasing the intrinsic sTEVp catalytic efficiency is likely to be challenging. The easiest approach to increase substrate cleavage may be to reduce the background activity, which would permit increased levels of sTEVp fragment expression (and thereby increase the amount of inducible TEVp). A previous study showed that a single point mutation to one of the fragments of a complemented protein, which was predicted to lower the affinity between the fragments, reduced background activity [37]. Perhaps a similar mutation would be successful with sTEVp.

An alternative split site of TEVp may be more suitable for complementation. Although the amount of C34V cleavage by 90/91 sTEVp was lower than 118/119 sTEVp (Fig. 2), the low background activity may allow methods that increase apparent/actual sTEVp concentration to compensate for the low catalytic efficiency. Alternative, untested, TEVp split sites also have the potential to lower affinity between fragments, although the obvious choices based on criteria proposed by Michnick *et al.* [38] have been tested in this investigation.

### Inducible TEVp using Fm proteins

The second method to develop an inducible TEVp using Fm proteins was, as yet, unsuccessful. This technique has potential advantages over protein fragment complementation in that it is reversible and rapid [24]. This approach is less disruptive to the TEVp active site than protein complementation, which may improve the catalytic efficiency of an inducible TEVp. Because of these advantages, occlusion of the active site by Fm moieties merits further investigation. Circular permutation of TEVp may uncover alternative N- and C- termini which, when bound to FM moieties, occlude the active site more effectively.

### Application of the sTEVp system to other target proteins

This investigation aimed to develop an inducible sTEVp with high catalytic efficiency and low background activity for single cell experiments, which may be inherently challenging. The variable nature of transfection leads to large differences in sTEVp expression levels from cell to cell and is likely to account for some of the high background activity and/or low substrate cleavage observed in these experiments. Despite this variability, a number of individual cells expressing pBud-sol-sTEVp had little background activity and good levels of C34V cleavage after 3 hours (Fig. 5B) and more than 50% Lyn-C34V cleavage in 20 minutes (Fig. 5C *ii* and S3 B *iii*). If the variability of expression levels could be better controlled, careful titration of the sTEVp pieces may provide the low background and efficient inducible protease activity necessary. It is difficult to predict how the inducible TEVp system would perform for other target proteins because the necessary level of inducible protease activity and acceptable level of background activity will be dependent on the aim and conditions of experiment. The level of inducible TEVp activity demonstrated in this study may be sufficient for other applications, especially experiments in which the cell-to cell variability poses less of a problem; for example, in cell lysates, where measurements are averaged from a large number of cells.

There are many applications for an inducible TEVp system for functional characterization of proteins. In particular, the ability to ablate the function of a chosen protein on a rapid scale is likely to be especially useful for the elucidation of a particular function of a protein that has multiple interrelated but temporally distinct roles. For example, investigation of the role of voltage-gated Ca^2+^-channel β-subunits in channel gating is impeded by the additional role of the β-subunit in trafficking the pore-forming α-subunit to the plasma membrane. The ability to abolish β-subunit function after it has completed its trafficking role is likely to provide considerable insights into its gating function.

If the level of inducible and background protease activity can be optimized, the sTEVp system provides a tool for studying protein function *in vivo*. Ideally, it would be possible to ablate endogenous protein function *in vivo* using an inducible, rationally designed protease selective for a chosen sequence. Unfortunately, the development of rationally designed proteases has been slow and insights into the mechanism of substrate specificity of TEVp have demonstrated the high complexity of this task [39]. In the absence of such a protease, TEVp has been used to investigate protein function *in vivo* in genetically malleable model organisms. In these systems, the endogenous protein was replaced by the protein containing a TRS: in *E. coli* [40] and *S. pombe* [41], a ‘knock-in’ technique was used to replace a chosen gene with the gene containing a TRS; in *Drosophila*, a mutant deficient in the gene of interest was rescued by transgenic expression of the gene containing a TRS [42]. In these examples, TEVp was expressed from a transgene under control of an inducible promoter. The application of similar knock-in/transgenic strategies for mammalian models is more challenging, but using these methods combined with the ability to express TEVp in particular tissues using cell-type specific promoters [42] and the temporal advantages of using sTEVp to control TEVp activity has the potential to provide a very powerful system to investigate protein function *in vivo*.

## Materials and Methods

### Cell culture

HEK-293 and HeLa cells (ATCC, Manassas, VA) were cultured at 37°C, 5% CO_2_ in Minimal Essential Medium (Invitrogen, Carlsbad, CA) supplemented with 10% fetal bovine serum (Hyclone, Logan, UT) and 100 units/ml penicillin, 100 µg/ml streptomycin (Invitrogen).

### Heterologous expression

For the FRET experiments, a suspension of approximately 5×10^5^ HeLa cells were transfected with plasmid DNA using fully de-acylated polyethylenimine [PEI; 43] at a nitrogen:phosphate of 6. Cells were added to a 12-well plate and incubated for overnight at 37°C, 5% CO_2_. 2 h before imaging, cells were replated onto 35 mm, collagen-coated, glass-bottomed petri dishes (Mattek, Ashland, MA).

For electrophysiological recordings, approximately 2×10^6^ HEK-293 cells were transfected using a pipette-based electroporation device (MicroPorator MP-100; Harvard Apparatus, Holliston, MA), according to manufacturer's instructions. Briefly, cells were resuspended in 50 µl of ‘R’ solution with 0.25–1 µg K_V_3.4 RNA, 1–3 µg sTEVp DNA and 0.1 µg pRFP-C1 DNA (as a tracer), as applicable. The electroporation parameters were 3 pulses of 1300 V, each pulse for a duration of 10 ms. Transfected cells were incubated overnight at 37°C, 5% CO_2_. 2 h prior to recording, cells were replated onto 35 mm plastic petri dishes.

### FRET studies

A widefield, inverted IX71 microscope (Olympus, Center Valley, PA), equipped with a PlanApo 60× 1.4 NA objective (Olympus), an X-cite Series 120PC mercury-halogen arc lamp (EXFO, Mississauga, ON, Canada) and a 12 Bit Retiga EXi CCD camera (QImaging, Surrey, BC, Canada) was used. Optical filters consisted of a dual CFP/YFP filter cube, CFP filter set (excitation 430/24 nm, emission 470/24 nm) and YFP filters (excitation 500/20 nm, emission 535/30 nm; all from Chroma, Rockingham, VT). The filters were mounted in filter wheels controlled by a Lambda 10–2 filter changer (Sutter, Novato, CA). Image acquisition and processing, camera control, and operation of the filter wheels were performed using custom-written IGOR Pro 6.0 (Wavemetrics, Lake Oswego, OR) procedures (S. Ikeda). Calculation of FRET efficiencies and FRET efficiency standards were described previously [20].

### Electrophysiology

Currents were recorded using conventional whole-cell patch-clamp techniques. Recordings were obtained using an Axopatch 200B amplifier (Molecular Devices, Sunnyvale, CA). Patch pipettes were fabricated from Corning 8250 borosilicate glass capillaries (World Precision Instruments, Sarasota, FL) using a P-97 Flaming-Brown micropipette puller (Sutter Instruments, Novato, CA). Pipettes were coated with Sylgard (Dow-Corning, Midland, MI) and fire-polished to a final resistance of approximately 2 ΜΩ when filled with internal solution. Voltage protocol generation and data acquisition were carried out using custom-designed software (S5) running on a Mac G4 computer (Apple, Cupertino, CA). Currents were filtered at 10 kHz using a four-pole low-pass Bessel filter, and digitized at 10 kHz. Uncompensated series resistance was <5 ΜΩ and was electrically compensated to approximately 90%. The external recording solution contained (in mM): 140 NaCl, 5.4 KCl, 10 HEPES, 0.8 MgCl_2_, 2 CaCl_2_, 15 Glucose. The pH was adjusted to 7.4 using NaOH. The pipette solution contained (in mM) 0.1 CaCl_2_, 11 EGTA, 10 HEPES, 120 potassium isethionate, 20 KCl, 4 MgATP, 0.1 Na_2_GTP. The pH was adjusted to 7.4 using KOH. The osmolality of the external and internal solutions were adjusted with sucrose to 315 and 290 mOsm, respectively. All recordings were carried out at room temperature (20–22°C).

A noninactivating current of <0.3 nA, likely to be caused by endogenous voltage-gated K^+^ or Cl^−^ channels [29], was elicited by the −80 to +50 mV step in untransfected HEK-293 cells (data not shown). For this reason, only currents with peak amplitudes >1.5 nA were used for calculations of inactivation.

### Molecular Biology

Standard PCR- and restriction-based cloning techniques were used in the construction of cDNA plasmids for this study. See “Supplemental methods S1” file for full details of the cDNA constructs. Briefly, the FRET based TEVp sensors were modifications of the previously described C32V construct [44]. Codon optimized TEVp (GenBank accession # DQ516974) was synthesized (Genescript, Piscataway, NJ) and cloned into a modified pCI (Promega, Madison, MI) expression vector. TEVp fragments were fused to the rapamycin binding partners FKBP12 (accession number U69485) and the FRB domain of FRAP1/mTOR (accession number NM_019906; residues 2018–2114) which have been described elsewhere [45]. Human K_V_3.4 (KCNC4; transcript variant 1; NM_004978) was amplified by the PCR from human whole-brain cDNA (BD Biosciences, Franklin Lakes, NJ). For RNA expression, K_V_3.4 constructs were sub-cloned into a custom vector containing a T7 promoter. RNA was transcribed from linearized plasmid DNA using mMESSAGE mMACHINE T7 kit (Ambion, Austin, TX) according to the manufacturer's instructions. The quality of the RNA was assessed on a gel and the concentration was determined by measuring the absorbance at 260 nm.

### Data analysis

Data were analyzed using IgorPro 6.0 and Prism 5.0 (GraphPad Software, San Diego, CA). Statistical comparisons were made using Kruskal-Wallis one-way ANOVA test with Dunn's post test, where appropriate. *P* values<0.05 were considered significant.

## Supporting Information

Supplemental Methods S1 (0.45 MB DOC)

Figure S1 Constructs were made containing TEVp fragments without the rapamycin-binding proteins. Scatter plot showing FRET efficiency measurements from cells transfected with 100 ng C34V and increasing amounts of 118/119 sTEVp constructs. # represents a significant difference of the median FRET efficiency between the control cells transfected with C34V alone (first column) and cells cotransfected with the mass of 118/119 sTEVp construct indicated. B) Time course of 118/119 sTEVp activity at 33°C. A plot showing the median FRET efficiencies of different cells transfected with 300 ng of 118/119 sTEVp (with FRB/FKBP12 domains) constructs at various time points after rapamycin addition. The time constant (τ) was calculated from a single-exponential curve fitted to the data (*n* = 23–29 cells at each time point).
(0.85 MB TIF)

Figure S2 Occlusion of the TEVp active site using the Fm mutant of FKBP12 was unsuccessful. A) Schematic diagram showing a potential method of controlling TEVp activity by rapamycin-dependent occlusion of the active site by Fm moieties. B) Fm-Cerulean and Fm-Venus plasmid constructs. C) Schematic diagram showing the rapamycin-dependent interaction of Fm-Venus and Fm-Cerulean. D) A scatter plot showing FRET efficiencies of different cells transfected with 100 ng of Fm-Venus and 100 ng of Fm-Cerulean without rapamycin, after exposure to 100 nM rapamycin for 3 hours, and 3 hours after the washout of rapamycin. E) Fm-TEVp plasmids. An FM moiety was fused to both ends of full-length TEVp and TEVp with N- and C-terminal truncations. The numbers correspond to the amino acid position on the full-length TEVp. F) Scatter plot showing FRET efficiencies measured from cells transfected with 100 ng of C34V alone and cells cotransfected with 300 ng of Fm-TEVp plasmids. # represents a significant difference of median FRET efficiency, in the absence of rapamycin, between control cells transfected with C34V alone (first column) and cells cotransfected with the indicated Fm-TEVp construct. * represents a significant difference between control cells (in the absence of rapamycin) transfected with the indicated Fm-TEVp construct and cells treated with 100 nM rapamycin for 3 hours. G) Constructs containing different linkers between the Fm and TEVp, and multiple copies of the Fm protein were tested. H) A scatter plot showing FRET efficiencies of different cells cotransfected with 100 ng C34V and 300 ng of Fm-TEVp plasmids. I) A schematic diagram showing attempts to block the active site with free Fm proteins and Fm-TEVp constructs. J) Scatter plot showing FRET efficiencies measured from cells cotransfected with 100 ng C34V and 300 ng of Fm-TEVp plasmids with (open circles) and without (closed circles) 300 ng of free Fm plasmid.
(1.60 MB TIF)

Figure S3 Time course of pBud-sTEVp activity in individual cells. A) Cells were transfected with 100 ng of soluble C34V alone (i), or cotransfected with either 1000 ng of pBud-sol-sTEVp (ii, iii) or 1000 ng pBud-mem-sTEVp (iv, v). FRET efficiencies were measured every 60 seconds for 20 minutes. Control recordings were made in the absence of rapamycin (ii, iv). For experiments in the presence of rapamycin (iii, v), 3 minutes of control measurements were made prior to the addition of 100 nM rapamycin. B) Cells transfected with 100 ng of mem-C34V. sTEVp constructs and rapamycin treatment are the same as (A).
(1.40 MB TIF)
