# Supplementary material for: Rapid Modification of Proteins Using a Rapamycin-Inducible Tobacco Etch Virus Protease System
Source: PLoS One. 2009 Oct 15;4(10):e7474. doi: 10.1371/journal.pone.0007474 (PMC2760398; doi:10.1371/journal.pone.0007474)
Supplement: Supplemental Methods S1 — (0.45 MB DOC) [file pone.0007474.s001.doc]

## Supplemental Materials: Methods

## A) FRET TEVp sensor constructs

***C32(M)V***

The BglII/EcoRI fragment of C32V was replaced with the following phosphorylated oligonucleotide duplex:

**5’-GATCT**GAGAACCTGTACTTCCAGATGCCGCGGG-3’

3’-**A**CTCTTGGACATGAAGGTCTACGGCGCCCTTAA-5’

(BglII-compatible end in bold type**,** TRS containing methionine residue at P1’ position (ENLYFQ**M**) in red, EcoRI-compatible overhang end)

***C34V***

The following phosphorylated oligonucleotide duplex was cloned into the BglII/AgeI sites of C32V:

**5’-GATCT**GAGAACCTGTACTTCCAGGGC*GATTATAAGGACGATGACGATAAGGAT*GGTGCTGGA-3’

3’-**A**CTCTTGGACATGAAGGTCCCG*CTAATATTCCTGCTACTGCTATTCCTA*CCACGACCTGGCC-5’

(BglII-compatible end in bold type, TRS (ENLYFQG) in red, FLAG® sequence (DYKDDDDKD) in italics, AgeI-compatible end underlined)

***mem-C34V***

The following phosphorylated oligonucleotide duplex was cloned into the NheI/BamH1 sites of C34V:

**5’-CTAGC**GCCACCATG*GGATGTATAAAATCAAAAGGGAAAGACAGCGCGGGAGCA*GGG-3’

3’-**G**CGGTGGTAC*CCTACATATTTTAGTTTTCCCTTTCTGTCGCGCCCTCGT*CCCCTAG-5’

(NheI-compatible end in bold type, Lyn11 sequence (GCIKSKGKDSAGA; from (Inoue *et al.*, 2005) in italics, BamHI-compatible overhang underlined)

## B) TEVp constructs

***TEVp***

The commercially synthesized TEVp was moved from pUC57 to a modified version of the mammalian expression vector ‘pCIK’ by restriction cloning using EcoRI and XbaI. pCIK, a modified pCI vector (Promega) was constructed by replacing the coding sequence for the ampicillin resistance gene in pCI with a Kanamycin resistance gene using a modification (Geiser *et al.*, 2001) of the QuickChange mutagenesis system (Stratagene, La Jolla, CA). Three mutations (T17S, N68D, I77V) proposed to increase solubility (van den Berg et al., 2006) were added to TEVp by QuickChange mutagenesis. Because of the origin of native TEVp peptide, the residue number of TEVp does not include the start ATG.

***RFP-TEVp***

TEVp protease was amplified from pCIK-TEVp using the primers shown below.

Forward: 5’-GATCGTCGACGGCGAGAGCCTGTTC-3’

(SalI site underlined, G1 of TEVp in red)

Reverse: 5’-GATCGGATCC*TTA*CCGGCGCCTCCT-3’

(BamHI site underlined, stop codon in italics, R241 of TEVp in red)

The PCR product was cloned into the SalI and BamHI sites of pRFP-C1.

## C) sTEVp with FRB/FKBP12 constructs

Initially, the coding sequence of the FRB domain of FKBP12-associated protein (FRAP; residues 2018‑2114) and FKBP12 were cloned into pVenus-N1 and pCerulean-N1 plasmid vectors, respectively. Sequences were generated using the PCR primers shown below.

‘pFRB-Venus’ (FRB cloned into pVenus-N1):

Forward: 5’-GATCAGATCTACC*ATG***GATATCCTCGAG**CGAGTAGCCATCCTCTGGCATG-3’

(BglII site underlined, start ATG in italics, EcoRV and XhoI sites in bold, R2018 of FRAP in red):

Reverse: 5’-GATCAAGCTTCTGCTTGGAGATCCGTCTGAAC-3’

(HindIII site underlined, R2114 of FRAP is in red)

The PCR product was cloned into the BglII and HindIII sites of pVenus-N1

‘pFKBP12-Cerulean’ (FKBP12 cloned into pCerulean-N1):

Forward: 5’-GATCAGATCTACC*ATG***GATATCCTCGAG**GGAGTGCAGGTGGAGACCATCTC-3’

(BglII site underlined, start codon in italics, EcoRV and XhoI sites in bold type, G2 of FKBP12 in red)

Reverse: 5’-GATCAAGCTTTTCCAGTTTTAGAAGCTCCAC-3’

(HindIII site underlined, E108 of FKBP12 in red)

The PCR product was cloned into the BglII and HindIII sites of pCerulean-N1

Following construction of pFRB-Venus and pFKBP12-Cerulean, the sTEVp plasmids were made using three different strategies:

***Strategy 1: 118/119 sTEVp***

The 118/119 split site was based on findings from Wehr *et al*., 2006. The 118/119 sTEVp plasmids constructed here, ‘pFRB-TEVp(1-118)’ and ‘pFKBP12-TEVp(1-119)’, were used as a basis for many of the other constructs used in this study.

pFRB-TEVp(1-118): The N-terminal fragment of sTEVp (residues 1-118 of TEVp) was cloned into pFRB-Venus**.**

1-118 of TEVp was amplified from pCIK-TEVp using the following primers.

Forward: 5’-GATCAAGCTTGGCGAGAGCCTGTTCAAGGGAC-3’

(HindIII site underlined, G1 of TEVp in red)

Reverse: 5’-GATCCCGCGGCT***TGTACA***GAGTCTGAAAGTTTGTGGTCACC-3’

(SacII site underlined, BsRGI site in bold type, T118 of TEVp in red)

The PCR product was cut with HindIII/SacII and cloned into pFRB-Venus plasmid vector at these sites. Venus was removed by digestion with BsrGI, which also cuts at the C-terminus of Venus.

pFKBP12-TEVp(1-119): The C-terminals fragment of sTEVp (residues 119-241 of TEVp) was cloned into pFKBP12-Cerulean

119-241 of TEVp was amplified from pCIK-TEVp using the following primers:

Forward: 5’-GATCAAGCTTAAGAGCATGTCCAGCATGGTG-3’

(HindIII site underlined, K119 of TEVp in red)

Reverse: 5’-GATCCCGCGGCT***TGTACA***GCCGGCGCCTCCTGCGATTCATC-3

(SacII site underlined, BsRGI site in bold type, R241 of TEVp in red)

The PCR product was cut with HindIII/SacII and cloned into pFKBP12-Cerluean plasmid vector at these sites. Cerulean was removed by digestion with BsrGI, which also cuts at the C-terminus of Cerulean.

***Strategy 2: 90/91, 136/137, 196/197 sTEVp***

Full-length TEVp was amplified from pCIK-TEVp using the following primers:

Forward: 5’-GATCAAGCTTGGCGAGAGCCTGTTCAAGGGAC-3’

(HindIII site underlined, G1 of TEVp in red)

Reverse: 5’-GATCCCGCGGCT***TGTACA***GCCGGCGCCTCCTGCGATTCATC-3

(SacII site underlined, BsRGI site in bold type, R241 of TEVp in red)

The full length TEVp PCR product was cut with HindIII/SacII and cloned into pFRB-Venus and pFKBP12‑Cerulean. The FP was removed from each construct by digestion with BsrGI (see above) to generate ‘pFRB-TEVp’ and ‘pFKBP12-TEVp’. Six deletion constructs were generated by QuickChange mutagenesis. For the N-terminal fragment TEVp (i.e. residues 1-90, 1-136, or 1-196 of TEVp) constructs, QuickChange Mutagenesis was performed on the pFRB-TEVp. For the C-terminal fragment TEVp, mutagenesis was performed on pFKBP12-TEVp. Only forward QuickChange primers are shown. The plasmid used in the mutagenesis and the new N- or C- terminus of the resulting TEVp fragment is displayed in parentheses.

sTEVp 90/91:

Forward: 5’-CATCATTAGGATGCCCAAGGACTAAAGCGGCCGCGACTCTAGATC-3’

(pFRB-TEVp, D90 of TEVp in red)

Forward: 5’-GGAGCTTCTAAAACTGGAAAAGCTTTTCCCCCCTTTTCCTCAG-3’

(pFKBP12-TEVp, F91 of TEVp in red)

sTEVp 136/137:

Forward: 5’-GTACCTTCCCATCATCTGACTAAAGCGGCCGCGACTCTAGATC-3’

(pFRB-TEVp, D136 of TEVp in red)

Forward: 5’-GGAGCTTCTAAAACTGGAAAAGCTTGGAATCTTCTGGAAGCACTG-3’

(pFKBP12-TEVp, G137 of TEVp in red)

sTEVp 196/197:

Forward: 5’-GACCAACCAGGAGGCCCAGTAAAGCGGCCGCGACTCTAGATC-3’

(pFRB-TEVp, Q196 of TEVp in red)

Forward: 5’-GGAGCTTCTAAAACTGGAAAAGCTTCAGTGGGTGAGCGGGTGG-3’

(pFKBP12-TEVp, Q197 of TEVp in red)

***Strategy 3: 96/97 sTEVp***

For the 96/97 sTEVp constructs, the N-terminal fragment of TEVp (residues 1-96) was fused to the N-terminus of FRB, in a TEVnt-FRB arrangement. For the other sTEVp constructs, the TEVp N-terminal fragments were fused to the C-terminus of FRB (see above).

‘pTEVp(1-96)-FRB’: The N-terminus fragment of sTEVp (residues 1-96 of TEVp) and FRB sequences were cloned into pcDNA3.1(+) (Invitrogen) by the following method.

TEVp residues 1-96 were amplified from pCIK-TEVp using the following primers:

Forward: 5’-GATCGCTAGCACC**ATG**GGCGAGAGCCTGTTCAAGG-3’

(NheI underlined, start ATG in bold type, G1 of TEVp in red)

Forward: 5’-GATTTCTAGATTAAAGCTTGTCGATATC*ACTACCTCCACCTCC*CTGAGGAAAAGGGGGGAAGTCC-3’

(XbaI/HindIII/EcoRV sites underlined, GGGS linker in italics, Q96 of TEVp in red)

The PCR product was cut with NheI and XbaI and cloned into these sites in pcDNA3.1, which created ‘pTEVp(1‑96)’. FRB was cut from the pFRB-Venus plasmid with EcoRV and HindIII and cloned into these sites of pTEVp(1-96).

‘pFKBP12-TEVp(97-241)’: The C-terminal fragment of sTEVp (residues 97-241 of TEVp) and FKBP12 were cloned into pcDNA3.1 by the following method.

TEVp residues 97-241 were amplified from pCIK-TEVp using the following primers:

Forward: 5’-GATCGCTAGCGGATCCGACAAGCTT*GGTGGAGGTGGAAGT*AAACTGAAGTTCCGAGAGCCCCAG-3’

(NheI/BamHI/HindIII sites underlined, G4S linker in italics, K97 in bold type)

Reverse: 5’-GATCTCTAGATTACCGGCGCCTCCTGCG-3’

(XbaI site underlined, R241 in red)

The PCR product was cut with NheI and XbaI and cloned into these sites in pcDNA3.1 which created ‘pTEVp(97-241)’.The FKBP12 sequence was cut from pFKBP12-Cerulean with BglII and HindIII and cloned into the BamHI/HindIII sites of pTEVp(97-241).

## D) sTEVp 118/119 without FRB/FKBP12

The FRB and FKBP12 domains were deleted from pFRB-TEVp(1-118) and pFKBP12-TEVp(119-241) plasmids (see Section C) using QuickChange mutagenesis with the primers shown below (only forward primers are displayed).

FRB deletion:

Forward: 5’-GGACTCAGATCTACC**ATG**GATATCGGCGAGAGCCTGTTCAAGG-3’

(Start codon in bold type and G1 of TEVp in red)

FKBP12 deletion:

Forward: 5’-GGACTCAGATCTACC**ATG**GATATCAAGAGCATGTCCAGCATGGTG-3’

(Start codon in bold type and K119 of TEVp in red)

## E) Unitary sTEVp constructs

All four unitary constructs were placed in a custom vector backbone, ‘pUHL’. This plasmid consisted of a pEGFP-N1 (Clontech) backbone with the multiple cloning sequence (MCS) and EGFP ORF and replaced by a custom MCS:


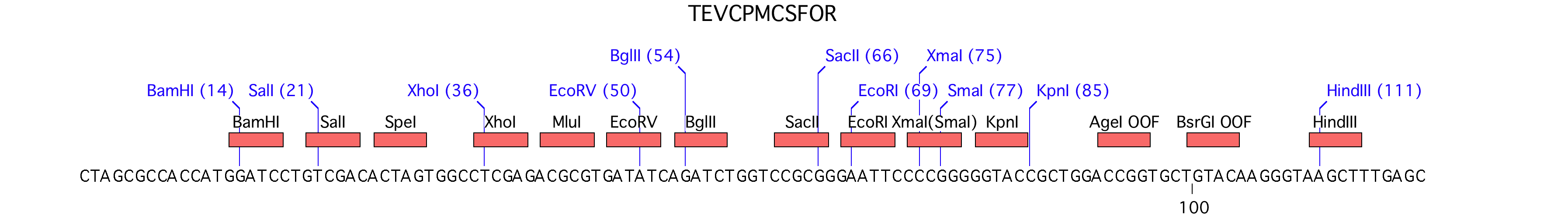


EGFP and the MCS were removed from pEGFP-N1 by cutting with NheI and NotI and replaced with the following phosphorylated oligonucleotide duplex containing NheI and NotI compatible overhangs and the custom MCS described above:

5’-CTAGC*GCCACCATGG*ATCCTGTCGACACTAGTGGCCTCGAGACGCGTGATATCAGATCTGGTCCGCGGGAATTCCCCGGGGGTA

3’-G*CGGTGGTAC*CTAGGACAGCTGTGATCACCGGAGCTCTGCGCACTATAGTCTAGACCAGGCGCCCTTAAGGGGCCCCCAT

CCGCTGGACCGGTGCTGTACAAGGGTAAGCTT**TGA**GC-3’

GGCGACCTGGCCACGACATGTTCCCATTCGAA**ACT**CGCCGG-5’

(Kozak sequence in italics, and the locations of restriction sites (not including the NheI-and NotI-compatible ends) are underlined, stop codon in bold type).

An α-helical linker, (EAAAR)_4_, was cloned into pUHL between the BglII and SacII restriction sites, using the following phosphorylated oligonucleotide duplex:

5’-**GATCT***GAAGCCGCAGCTAGAGAGGCTGCAGCCAGGGAAGCAGCTGCCAGAGAGGCAGCTGCAAGG*CCGC-3’

3’-**A**C*TTCGGCGTCGATCTCTCCGACGTCGGTCCCTTCGTCGACGGTCTCTCCGTCGACGTTCC*GG-5’

(BglII-compatible end in bold type, SacII-compatible end underlined, (EAAAR)_4_ in italics underlined)

Cassettes for the four unitary STEVp constructs were amplified using the sTEVp plasmids and the primer sets shown below. The PCR products cut and inserted into the appropriate restriction sites in pUHL‑(EAAAR)_4_ plasmid.

***Unitary sTEVp 1***


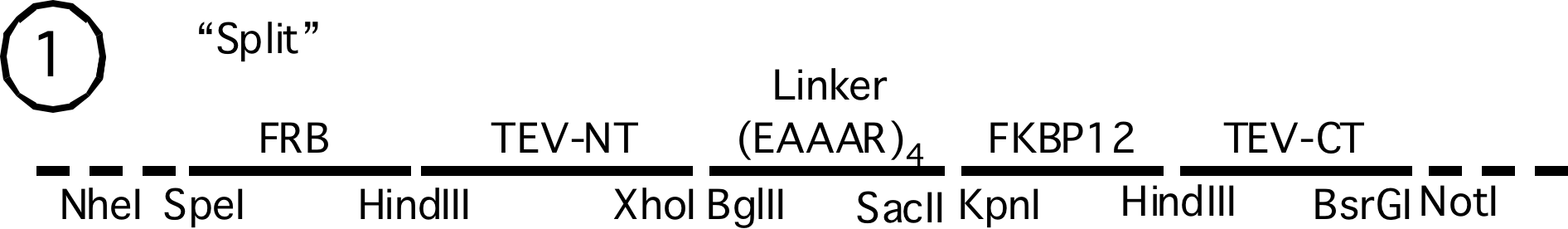


FRB-TEVp(1-118) was amplified from pFRB-TEVp(1-118) (Section C) using the following primers:

Forward: 5’-GATCACTAGTCGAGTAGCCATCCTCTGG-3’

(SpeI site underlined, R2 of FRB in red)

Reverse: 5’-GATCCTCGAGAGTCTGAAAGTTTGTGGTCACC-3’

(Xho1 site underlined, T118 of TEVp in red)

FKBP12-TEVp(119-241) amplified from pFKBP12-TEVp( 1-119) (Section C) using the following primers:

Forward: 5’-GATCGGTACCGGAGTGCAGGTGGAGACC-3’

(KpnI site underlined, G2 of FKBP12 in red)

Reverse: 5’-GATCGCGGCCGC*TCA*CT**TGTACA**GCCGGCGCCTCCTGCGATTCATC-3’

(BsrGI site is underlined, stop codon in italics, and BsrGI site in bold type, R241 of TEVp in red)

***Unitary sTEVp 2***


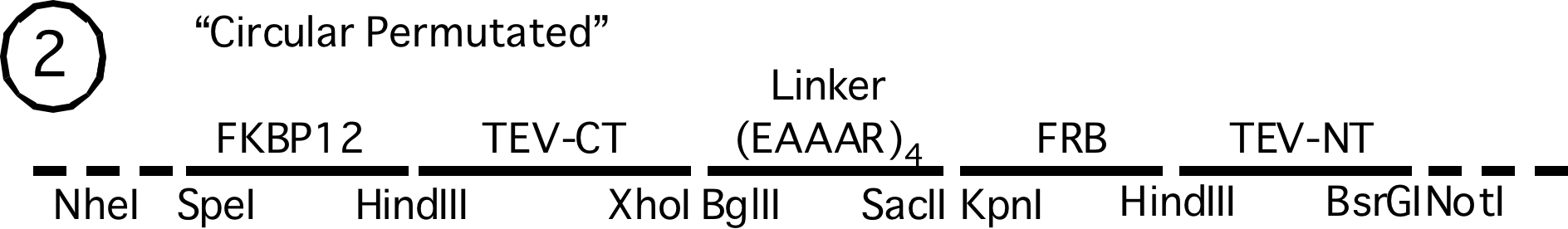


FKBP12-TEVp(119-241) amplified from pFKBP12-TEVp(1-119) using the following primers:

Forward: 5’-GATCACTAGTGGAGTGCAGGTGGAGACC-3’

(SpeI site underlined, G2 of FKBP12 in red)

Reverse: 5’-GATCCTCGAGCCGGCGCCTCCTGCGATTCATC-3’

(XhoI site underlined, R241 of TEVp in red)

FRB-TEVp(1-118) was amplified from pFRB-TEVp(1-118) using the following primers:

Forward: 5’-GATCGGTACCCGAGTAGCCATCCTCTGG-3’

(KpnI site underlined, R2018 of FRAP in red)

Reverse: 5’-GATCGCGGCCGC*TCA*CT**TGTACA**GAGTCTGAAAGTTTGTGGTCACC-3’

(NotI site underlined, stop codon in italics, BsrGI in bold type, T118 of TEVp in red )

***Unitary sTEVp 3***


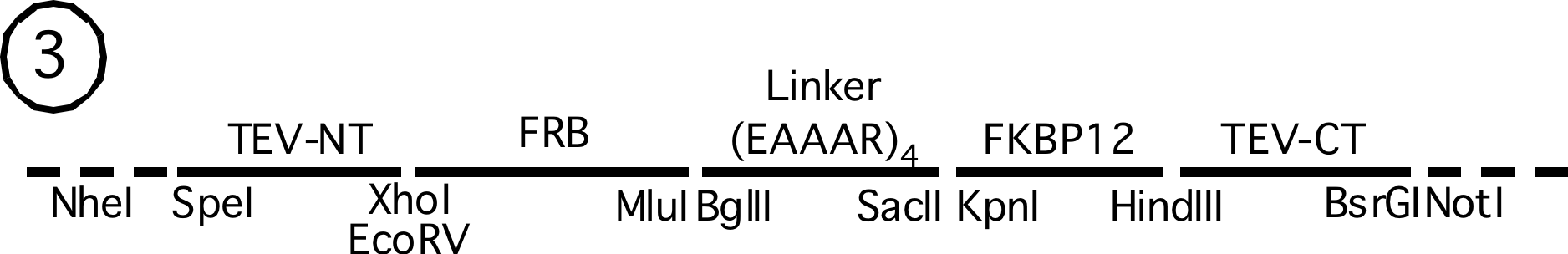


‘pTEVp(1-118)-FRB’, a plasmid containing residues 1-118 of TEVp fused to the N-terminus of FRB, was generated by insertion of TEVp residues 97-118 into the pTEVp(1‑96)‑FRB plasmid (see Section C, 96/97 sTEVp) using QuickChange mutagenesis with the following primers (only forward primer shown).

Forward : 5’-GGACTTCCCCCCTTTTCCTCAGAAACTGAAGTTCCGAGAGCCCCAGAGAGAGGAGAGAATCTGTCTGGTGACCAC

AAACTTTCAGACTGGAGGTGGAGGTAGTGATATCC-3’

(K97 to T118 of TEVp underlined)

The TEVp(1-118)-FRB sequence was then amplified from pTEVp(1-118)-FRB using the following primers:

Forward: 5’-GATCACTAGTGGCGAGAGCCTGTTCAAGG-3’

(SpeI site underlined, G1 of TEVp in red)

Reverse: 5’-GATCACGCGTCTGCTTGGAGATCCGTCTG-3’

(MluI site underlined, Q2114 of FRAP in red)

The FKBP12-TEVp(119-241) sequence was amplified from pFKBP12-TEVp(119-241) using the appropriate primers described in ‘Unitary sTEVp 1’.

***Unitary sTEVp 4***


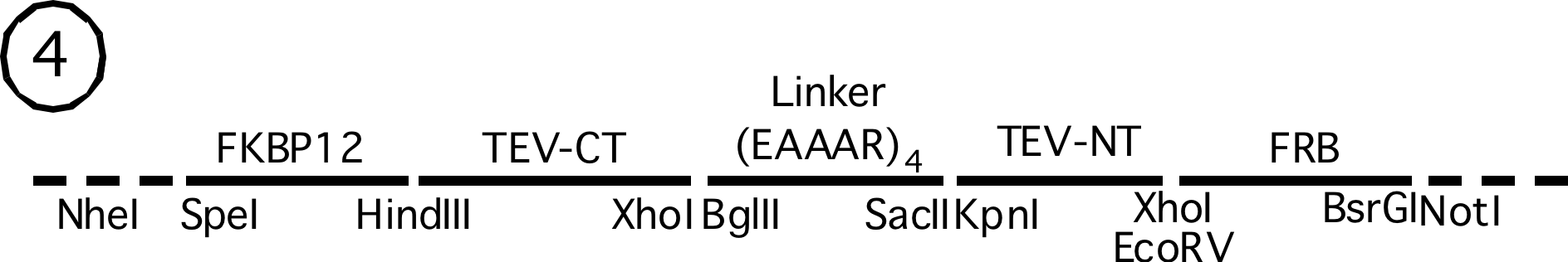


FKBP12-TEVp(119-241) sequence was amplified from pFKBP12-TEVp(119-241) using the appropriate primers described in ‘Unitary sTEVp 2’

TEVp(1-118)-FRB sequence was amplified from pTEVp(1-118)-FRB (plasmid generated during the construction of ‘Unitary sTEVp 3’) using the following primers:

Forward: 5’-GATCGGTACCGGCGAGAGCCTGTTCAAGG-3’

(KpnI site underlined, G1 of TEVp in red)

Reverse: 5’-GATCGCGGCCGC*TCA*CT**TGTACA**GCTGCTTGGAGATCCGTCTG-3

(NotI site underlined, stop codon in italics, BsrGI in bold type, and Q2114 of FRAP in red)

## F. cis-based sTEVp constructs

***C34V-FRB-TEVnt***

FRB-TEVp(1-118) was amplified from pFRB-TEVp(1-118) (see Section C) using the following primers:

Forward: 5’-GAGCTGTACAAG**GGTGGA**CGAGTAGCCATCCTCTGG-3’

(BsrGI site underlined, GG linker in bold, and R2018 of FRAP in red)

Reverse: 5’-GATCGCGGCCGC*TCA*AGTCTGAAAGTTTGTGGTCAC-3’

(NotI site underlined, stop codon in italics, and T118 of TEVp in red)

The PCR product was cloned into the BsrGI and NotI sites of C34V construct described in Section A. The second sTEVp fragment, FKBP12-TEVp(119-241), was expressed from pFKBP12-TEVp(119-241), described in Section C.

***C34V-FKBP12-TEVct***

FKBP12-TEVp(119-241) was amplified from pFKBP12-TEVp(119-241) (Section C) using the following primers:

Forward: 5’-GAGCTGTACAAG**GGTGGA**GGAGTGCAGGTGGAGACC-3’

(BsrGI site underlined, GG linker in bold, G2 of FKBP12 in red)

Reverse: 5’-GATCGCGGCCGC*TCA*CCGGCGCCTCCTGCGATTC-3’

(NotI site underlined, stop codon in italics, R241 in red)

The product was cloned into the BsrGI and NotI sites of the C34V construct described in Section A. The second TEVp fragment, FRB-TEVp(1-118), was expressed from pFRB-TEVp(1-118), described in Section C.

## F. pBud-sTEVp constructs

***pBud-sol-sTEVp***

The pBudCE4.1 plasmid (Invitrogen) contains two promoters, CMV and EF1-α, which were used to drive expression of FRB-TEVp(1-118) and FKBP12-TEVp(119-241), respectively. Insertion of the sTEVp fragments was carried out in two steps. First, FKBP12-TEVp(119-241) was excised from pFKBP12‑TEVp(119-241) (see Section C) using BglII and NotI and the resulting overhangs filled. This excised piece of pFKBP12‑TEVp(119-241) inserted into pBudCE4.1 that had been cut with XhoI and the resulting overhangs filled. Secondly, FRB-TEVp(1-118) was excised from pFRB-TEVp(1-118) (see Section C) using BglII and NotI. The overhangs of the excised portion were filled. The sequence was inserted into pBudCE4.1 containing FKBP12-TEVp(119-241), which had been cut with XbaI and the overhangs filled.

***pBud-mem-sTEVp***

A *lyn11* sequence was inserted at the N-terminus of FKBP12-TEVp(119-241) cassette in pBud-sol-sTEVp plasmid by QuickChange mutagenesis with the following primer set (only forward primer is shown):

5’-GGTACCAGCACAGTGGACTCACCATG**GGATGTATAAAATCAAAAGGGAAAGACAGCGCGGGAGCA**GATATCGGAGTGCAG

GTGGAGACCATCTC-3’

(Lyn11 sequence, GCIKSKGKDSAGA, is shown in bold-type, EcoRV site underlined, G2 of FKBP12 in red)

## G) K_V_3.4 constructs

***Wild-type K_V_3.4***

Human K_V_3.4 (KCNC4; transcript variant 1; NM_004978) was amplified from human whole-brain cDNA (BD Biosciences, Franklin Lakes, NJ) with the following primers:

Forward: 5’-GATCGAATTCGCCACC*ATG*ATCAGCTCGGTGTGTGTCTC-3’

(EcoRI site underlined, start codon in italics, I2 of K_V_3.4 in red)

Reverse: 5’-GATC**GCGGCCGC***TCA*ATGTGGCAGGAACAGAGTC-3’

(NotI site underlined, stop codon in italics, H635 of K_V_3.4 in red)

The PCR product was cloned into PCR-Blunt II-Topo vector (Invitrogen). The K_V_3.4 sequence was then sub-cloned into the EcoRI and NotI sites of pCIK vector (see Section B). The resulting plasmid was termed ‘pCIK-K_V_3.4’.

***EGFP-K_V_3.4***

The EGFP-K_V_3.4 fusion construct consists of an EGFP ORF linked by a TRS to the N-terminus of K_V_3.4. The following primers were used to amplify Kv3.4 from pCIK-K_V_3.4 (above):

Forward: 5’-GATCGAATTCA**GAGAACCTGTACTTCCAGGGC**ATCAGCTCGGTGTGTGTCTC-3’

(EcoRI site underlined, the TRS in bold-type, I2 of K_V_3.4 in red)

Reverse: 5’-GATCGTCGAC*TCA*ATGTGGCAGGAACAGAGTC-3’

(SalI site underlined, stop codon in italics, H635 of K_V_3.4 in red)

The PCR product was cloned into the EcoRI and SalI site of pEFGP-C1.

For *in vitro* transcription, the EGFP-Kv3.4 sequence was sub-cloned from pEGFP-C1 vector above into a custom RNA vector, ‘pRNA’, created as follows:

The mammalian expression vector pCIK (Section B) was modified by replacing the native MCS with the MCS and EGFP ORF from pEGFP-N1 (Clontech) using restriction digestion of both plasmids with NheI and NotI.

The following phosphorylated oligonucleotide duplex containing the human β-globin 3’UTR (NM_000518, bases 494-626) was inserted into the modified pCIK at the NotI site:

**5’-GGCC**GC*AGCTCGCTTTCTTGCTGTCCAATTTCTATTAAAGGTTCCTTTGTTCCCTAAGTCCAACTACTAAACTGGGGGATATTA*

3’-CGTCGAGCGAAAGAACGACAGGTTAAAGATAATTTCCAAGGAAACAAGGGATTCAGGTTGATGATTTGACCCCCTATAAT

*TGAAGGGCCTTGAGCATTTGGATTCTGCCTAATAAAAAACATTTATTTTCATTGC*GTTTAAAC-3’

ACTTCCCGGAACTCGTAAACCTAAGACGGATTATTTTTTGTAAATAAAAGTAACGCAAATTTG**CCGG**-5’

(NotI compatible overhangs in bold type, 3’ NotI site, red, is not regenerated; PmeI site underlined, 3’ UTR of β-globin in italics)

A second human β-globin 3’UTR was then inserted at the PmeI site using the following phosphorylated oligonucleotide duplex:

5’-*AGCTCGCTTTCTTGCTGTCCAATTTCTATTAAAGGTTCCTTTGTTCCCTAAGTCCAACTACTAAACTGGGGGATATTATGAAGG*

3’-TCGAGCGAAAGAACGACAGGTTAAAGATAATTTCCAAGGAAACAAGGGATTCAGGTTGATGATTTGACCCCCTATAATACTTCC

*GCCTTGAGCATTTGGATTCTGCCTAATAAAAAACATTTATTTTCATTGC***ATTTAAAT**GTTTAAAC-3’

CGGAACTCGTAAACCTAAGACGGATTATTTTTTGTAAATAAAAGTAACG**TAAATTTA**CAAATTTG-5’

(3’ UTR of β-globin in italics, SwaI site in bold type, PmeI site underlined)

The T7 promoter was optimized by QuickChange mutagenesis based on the sequence requirements recommended by the mMESSAGE mMACHINE™ T7 Ultra protocol (Applied Biosystems). The following primers were used (only forward primer shown)

5’-GAGTACT**TAATACGACTCACTATAGGGAGA**GATATCACGCGTTCTAGAGCTAGCGCTACCGGACTCAG-3’

(T7 promoter in bold type, EcoRV, MluI (AflIII), XbaI, NheI sites underlined).

Finally, BamHI, BglII and HindIII sites were removed from the vector. The sites were sequentially removed by single point mutations using QuickChange mutagenesis. The following primers were used (only forward sequence is shown):

BamHI: 5’-AATCGATAAGGATC**A**GGGCTGGCGTAATAGC-3’

BglII: 5’-TCACATGGCTCGACAGATC**A**TCAATATTGGCCATTAGCC-3’

HindIII: 5’-CCGTCAGATCACTAGA**C**GCTTTATTGCGGTAG-3’

(Restriction sites underlined, point mutation in bold type)

## H. Fm-FP and Fm-TEVp constructs (Supplemental Fig. 3)

***FmC and FmV***

FKBP12 was cloned into pCerulean-N1 and pVenus-N1 as described in Section C. ‘Fm’, the homodimerizing FKBP12 protein containing a F36M mutation, was generated using QuickChange mutagenesis using primers below (only forward primer shown):

Forward: 5’-GCTTGAAGATGGGAAGAAAATGGACTCCTCTCGGGACAG-3’

(F36M mutation underlined)

***FmT***

A concatemer of 2 Fm sequences was inserted in pUHL plasmid (Section E). The Fm ORF was amplified from the Fm-C vector with the following primers and cloned sequentially into the appropriate sites:

Forward: 5’-GATCACTAGTGGAGTGCAGGTGGAGACCATCTC-3’

(SpeI site underlined, G2 of Fm in red)

Reverse: 5’-GATCCTCGAGTTCCAGTTTTAGAAGCTCCAC-3

(XhoI site underlined, E108 of Fm in red)

Forward: 5’-GATCGGTACCGGAGTGCAGGTGGAGACCATCTC-3’

(KpnI site underlined, G2 of Fm in red)

Reverse: 5’-GATCAAGCTTTTCCAGTTTTAGAAGCTCCAC-3’

(HindIII site underlined, E108 of Fm in red)

Full length TEVp was amplified from pCIK-TEVp (Section B) with the following primers. The PCR product was then cloned into the MluI and EcoRI sites (between the Fm sequences).

Forward: 5’-GATCACGCGTGGCGAGAGCCTGTTCAAGG-3’

(MluI site underlined, G1 of TEVp in red)

Reverse: 5’-GATCGAATTCCCGGCGCCTCCTGCGATTC-3’

(EcoRI site underlined, R241 of TEVp in red)

***FmTΔN, FmTΔC, and FmTΔNΔC,***

N- or C-terminal truncated TEVp was amplified from pCIK-TEVp using the appropriate primer used for the full-length Fm-TEVp construct (FmT, above) and one of the truncation primers shown below. For FmTΔNΔC, both truncation primers were used. The PCR product was then cloned into the MluI and EcoRI sites into the same pUHL vector containing 2 Fm sequences, as described as for FmT.

FmTΔN:

Forward: 5’-GATCACGCGTGACTACAACCCTATCAGTAGC-3’

(MluI site underlined, D10 of TEVp in red)

(Reverse primer from FmT)

FmTΔC:

(Forward primer from FmT):

Reverse: 5’-GATCGAATTCCATAAACACCTTGTGCCC-3’

(EcoRI site underlined, M218 of TEVp in red)

***Additional Fm-TEVp constructs***

Linkers and additional copies of FM were added to the above FmT construct to generate the further Fm‑TEVp constructs. Linkers were added sequentially at the XhoI/MluI site 5’ to the TEVp sequence and to the EcoRI/XmaI site 3’ to the TEVp sequence. Oligonucleotides were designed with overhangs as shown.

Phosphorylated oligonucleotide duplex containing (GGGGS)_4_ linker with XhoI and MluI sites:

5’-**TCGAG***GGTGGCGGAGGCTCCGGAGGTGGAGGCTCTGGTGGCGGAGGCTCCGGAGGTGGAGGCTCT*A-3’

3’-**C***CCACCGCCTCCGAGGCCTCCACCTCCGAGACCACCGCCTCCGAGGCCTCCACCTCCGAGA*TGCGC-5’

(XhoI-compatible end in bold, (GGGGS)_4_ in italics, MluI-compatible end underlined)

Phosphorylated oligonucleotide duplex containing (GGGGS)_4_ linker with EcoRI and XmaI sites:

5’-**AATTC***GGTGGCGGAGGCTCCGGAGGTGGAGGCTCTGGTGGCGGAGGCTCCGGAGGTGGAGGCTCT*C-3’

3’-**G***CCACCGCCTCCGAGGCCTCCACCTCCGAGACCACCGCCTCCGAGGCCTCCACCTCCGAGA*GGGCC-5’

(EcoRI-compatible end in bold, (GGGGS)_4_ in italics, XmaI-compatible end underlined)

Phosphorylated oligonucleotide duplex containing (EAAAR)_4_ linker with XhoI and MluI sites:

5’-**TCGAG***GAAGCCGCAGCTAGAGAGGCTGCAGCCAGGGAAGCAGCTGCCAGAGAGGCAGCTGCAAGG*A-3’

3’-**C**CTTCGGCGTCGATCTCTCCGACGTCGGTCCCTTCGTCGACGGTCTCTCCGTCGACGTTCCTGCGC-5’

(XhoI-compatible end in bold, (EAAAR)_4_ in italics, MluI-compatible end underlined)

Phosphorylated oligonucleotide duplex containing (EAAAR)_4_ linker with EcoRI and XmaI sites:

5’-**AATTC***GAAGCCGCAGCTAGAGAGGCTGCAGCCAGGGAAGCAGCTGCCAGAGAGGCAGCTGCAAGG*C-3’

3’-***G****CTTCGGCGTCGATCTCTCCGACGTCGGTCCCTTCGTCGACGGTCTCTCCGTCGACGTTCC*GGGCC-5’

(EcoRI-compatible end in bold, (EAAAR)_4_ in italics, XmaI-compatible end underlined)

For the Fm2E-TEV-2E-Fm construct, a second pair of (EAAAR)_4_ linkers was inserted using the duplexes shown below.

Phosphorylated oligonucleotide duplex containing (EAAAR)_4_ linker containing one non-regenerative MluI site and one regenerative MluI site:

5’-**CGCGC***GAAGCCGCAGCTAGAGAGGCTGCAGCCAGGGAAGCAGCTGCCAGAGAGGCAGCTGCAAGG*A

3’-**G***CTTCGGCGTCGATCTCTCCGACGTCGGTCCCTTCGTCGACGGTCTCTCCGTCGACGTTCC*TGCGC

(Non-regenerative MluI-compatible end in bold, (EAAAR)_4_ in italics, regenerative MluI-compatible end underlined)

Phosphorylated oligonucleotide duplex containing (EAAAR)_4_ linker containing one non-regenerative XmaI site and one regenerative XmaI site:

5’-**CCGGA***GAAGCCGCAGCTAGAGAGGCTGCAGCCAGGGAAGCAGCTGCCAGAGAGGCAGCTGCAAGG*C-3’

3’-**T***CTTCGGCGTCGATCTCTCCGACGTCGGTCCCTTCGTCGACGGTCTCTCCGTCGACGTTCC*GGGCC-5’

(Non-regenerative XmaI-compatible end in bold, (EAAAR)_4_ in italics, regenerative XmaI compatible end underlined)

Additional copies of Fm were amplified from the FmC plasmid and inserted into the Fm-TEVp constructs as required. The SalI and SpeI restriction sites were used for the N-terminus Fm insertion, and the HindIII site and NotI sites were used for the C-terminus Fm insertion. Amplification was carried out using the PCR primer sets shown below.

For the N–terminal Fm insert:

Forward: 5’-GATCGTCGACGGAGTGCAGGTGGAGACCATCTC-3’

(SalI site underlined, G2 of Fm in red)

Reverse: 5’-GATCACTAGTTTCCAGTTTTAGAAGCTCCAC-3’

(SpeI site underlined, E108 of Fm in red)

For the C–terminal Fm insert:

Forward: 5’-GATCAAGCTTGGAGTGCAGGTGGAGACCATCTC-3’

(HindIII site underlined, G2 of Fm in red)

Reverse: 5’-GATCGCGGCCGC*TCA*TTCCAGTTTTAGAAGCTCCAC-3’

(NotI site underlined, stop codon in italics, E108 of Fm in red)

## Supplemental Material: References

1 Inoue T, Heo WD, Grimley JS, Wandless TJ, Meyer T (2005) An inducible translocation strategy to rapidly activate and inhibit small GTPase signaling pathways. Nat Methods 2: 415-418.

2 Geiser M, Cebe R, Drewello D, Schmitz R (2001) Integration of PCR fragments at any specific site within cloning vectors without the use of restriction enzymes and DNA ligase. Biotechniques 31: 88-92.

3 van den Berg S, Lofdahl PA, Hard T, Berglund H (2006) Improved solubility of TEV protease by directed evolution. J Biotechnol 121: 291-298.

4 Wehr MC, Laage R, Bolz U, Fischer TM, Grunewald S, Scheek S, Bach A, Nave KA, and. Rossner MJ (2006) Monitoring regulated protein-protein interactions using split TEV. Nat Methods 3: 985-993.
