# Supplementary figures and images for: Rapid Modification of Proteins Using a Rapamycin-Inducible Tobacco Etch Virus Protease System
Source: PLoS One. 2009 Oct 15;4(10):e7474. doi: 10.1371/journal.pone.0007474 (PMC2760398; doi:10.1371/journal.pone.0007474)

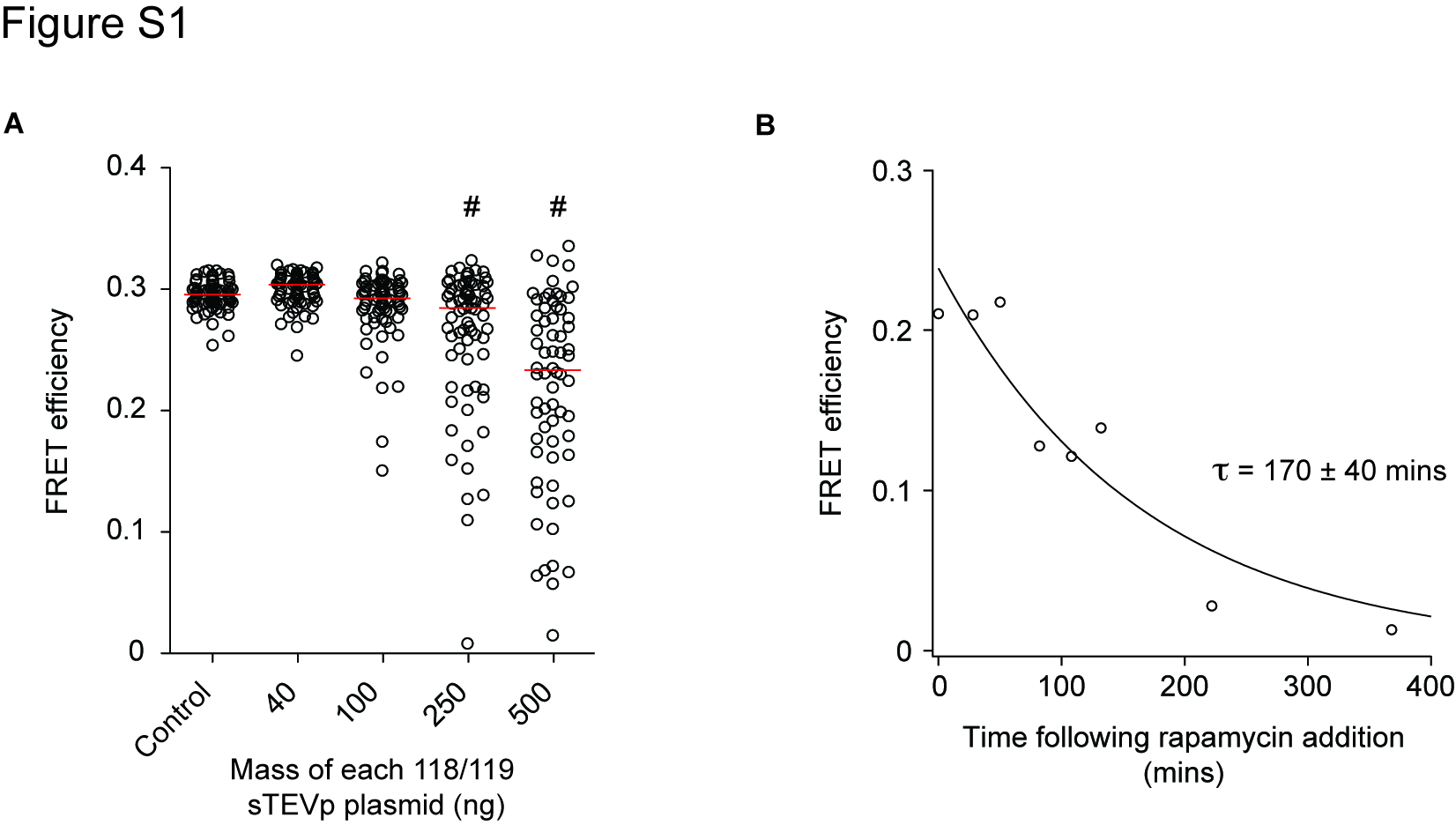

Supplement: Figure S1 — Constructs were made containing TEVp fragments without the rapamycin-binding proteins. Scatter plot showing FRET efficiency measurements from cells transfected with 100 ng C34V and increasing amounts of 118/119 sTEVp constructs. # represents a significant difference of the median FRET efficiency between the control cells transfected with C34V alone (first column) and cells cotransfected with the mass of 118/119 sTEVp construct indicated. B) Time course of 118/119 sTEVp activity at 33°C. A plot showing the median FRET efficiencies of different cells transfected with 300 ng of 118/119 sTEVp (with FRB/FKBP12 domains) constructs at various time points after rapamycin addition. The time constant (τ) was calculated from a single-exponential curve fitted to the data (n = 23–29 cells at each time point). (0.85 MB TIF) [file pone.0007474.s002.tif]

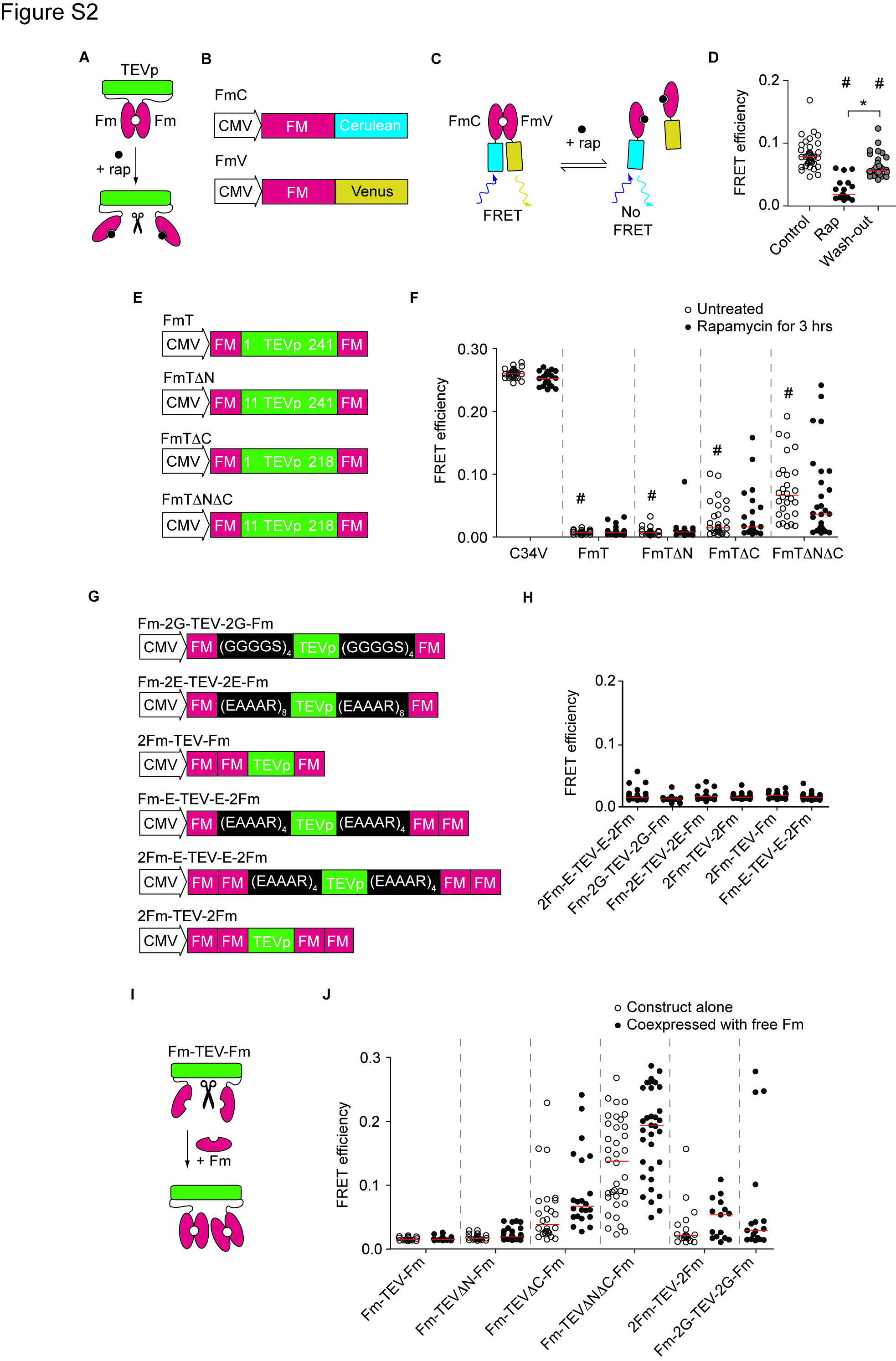

Supplement: Figure S2 — Occlusion of the TEVp active site using the Fm mutant of FKBP12 was unsuccessful. A) Schematic diagram showing a potential method of controlling TEVp activity by rapamycin-dependent occlusion of the active site by Fm moieties. B) Fm-Cerulean and Fm-Venus plasmid constructs. C) Schematic diagram showing the rapamycin-dependent interaction of Fm-Venus and Fm-Cerulean. D) A scatter plot showing FRET efficiencies of different cells transfected with 100 ng of Fm-Venus and 100 ng of Fm-Cerulean without rapamycin, after exposure to 100 nM rapamycin for 3 hours, and 3 hours after the washout of rapamycin. E) Fm-TEVp plasmids. An FM moiety was fused to both ends of full-length TEVp and TEVp with N- and C-terminal truncations. The numbers correspond to the amino acid position on the full-length TEVp. F) Scatter plot showing FRET efficiencies measured from cells transfected with 100 ng of C34V alone and cells cotransfected with 300 ng of Fm-TEVp plasmids. # represents a significant difference of median FRET efficiency, in the absence of rapamycin, between control cells transfected with C34V alone (first column) and cells cotransfected with the indicated Fm-TEVp construct. * represents a significant difference between control cells (in the absence of rapamycin) transfected with the indicated Fm-TEVp construct and cells treated with 100 nM rapamycin for 3 hours. G) Constructs containing different linkers between the Fm and TEVp, and multiple copies of the Fm protein were tested. H) A scatter plot showing FRET efficiencies of different cells cotransfected with 100 ng C34V and 300 ng of Fm-TEVp plasmids. I) A schematic diagram showing attempts to block the active site with free Fm proteins and Fm-TEVp constructs. J) Scatter plot showing FRET efficiencies measured from cells cotransfected with 100 ng C34V and 300 ng of Fm-TEVp plasmids with (open circles) and without (closed circles) 300 ng of free Fm plasmid. (1.60 MB TIF) [file pone.0007474.s003.tif]

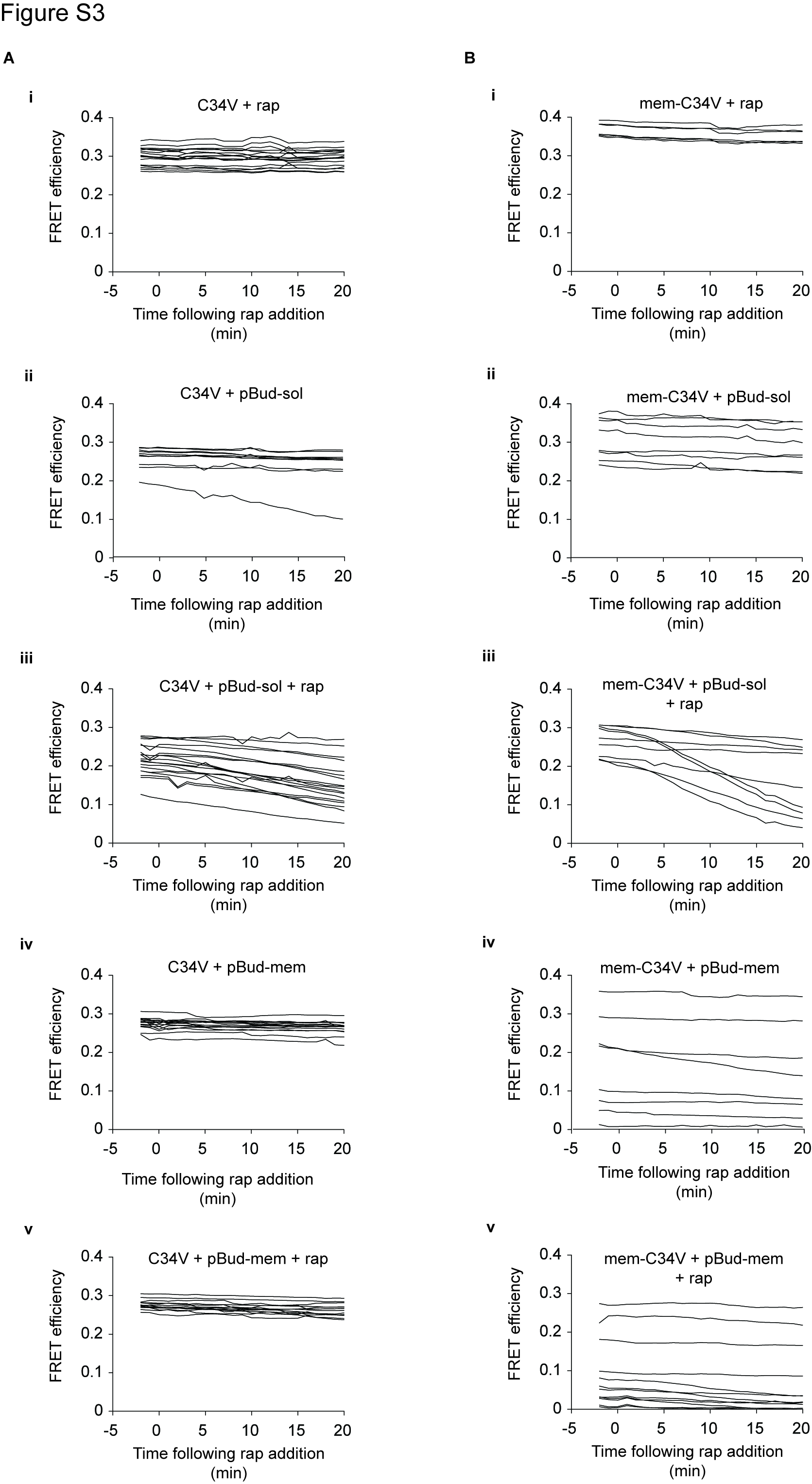

Supplement: Figure S3 — Time course of pBud-sTEVp activity in individual cells. A) Cells were transfected with 100 ng of soluble C34V alone (i), or cotransfected with either 1000 ng of pBud-sol-sTEVp (ii, iii) or 1000 ng pBud-mem-sTEVp (iv, v). FRET efficiencies were measured every 60 seconds for 20 minutes. Control recordings were made in the absence of rapamycin (ii, iv). For experiments in the presence of rapamycin (iii, v), 3 minutes of control measurements were made prior to the addition of 100 nM rapamycin. B) Cells transfected with 100 ng of mem-C34V. sTEVp constructs and rapamycin treatment are the same as (A). (1.40 MB TIF) [file pone.0007474.s004.tif]
